# Supplementary material for: Restorative Community Building Practices: A Train-the-Trainer Workshop for Medical Students, Staff, and Faculty
Source: MedEdPORTAL. 2025 Sep 23;21:11547. doi: 10.15766/mep_2374-8265.11547 (PMC12454668; doi:10.15766/mep_2374-8265.11547)
Supplement: Supplementary file 1 — Training Schedule.docxRP Training Lecture 1.pptxRP Training Circle Scripts.docxRP in Academic Medicine.docxRP Training Lecture 2.pptxWorkshop Pre- and Postsurveys.docx3-Month Follow-Up Survey.docx [file mep_2374-8265.11547-s001.zip › A. Training Schedule.docx]

**Appendix A: Workshop Schedule**

Purpose: This appendix describes the schedule for the workshop with suggested time allocations for each activity.

8:30 – 9:00 am: Introduction to RJ concepts

9:00 – 9:15 am: Large Group Circle Activity

9:15 - 10:00 am: Break into 4 small groups for community building circle

10:00 – 10:15 am: Break

10:15 – 11:00 am: Debrief on circle and lecture on circling approach and practices

11:00 – 12:00 pm: Introduction to restorative practices – affective statements/ affective questions

12:00 – 1:00 pm: Lunch with lecture discussing RJ theory

1:00 – 1:30 pm: Break into 3-person groups and design circle scripts using worksheet

1:30 – 2:45 pm: Combine into groups of 6. First group leads circle they designed*

2:45 – 3:00 pm: Debrief on leading first circle

3:00 – 4:00 pm: Switch keepers to lead second circle

4:00 – 4:30 pm: Debrief on circling process and training

4:30 – 5:00 pm: Plan individual RJ “intervention” with support from trainers

* After the 3-person groups completed their scripts, each 2 groups were combined to create circle groups of 6 participants each. Participants in each circle group took turns leading 2 circles, using the scripts they designed. Since each script was developed by 3 individuals, groups could delegate the entire circle facilitation to one person or take turns to facilitate different portions of their scripts.
