# Supplementary material for: Restorative Community Building Practices: A Train-the-Trainer Workshop for Medical Students, Staff, and Faculty
Source: MedEdPORTAL. 2025 Sep 23;21:11547. doi: 10.15766/mep_2374-8265.11547 (PMC12454668; doi:10.15766/mep_2374-8265.11547)
Supplement: Supplementary file 1 — Training Schedule.docxRP Training Lecture 1.pptxRP Training Circle Scripts.docxRP in Academic Medicine.docxRP Training Lecture 2.pptxWorkshop Pre- and Postsurveys.docx3-Month Follow-Up Survey.docx [file mep_2374-8265.11547-s001.zip › B. RP Training Lecture 1.pptx]

## Slide 1
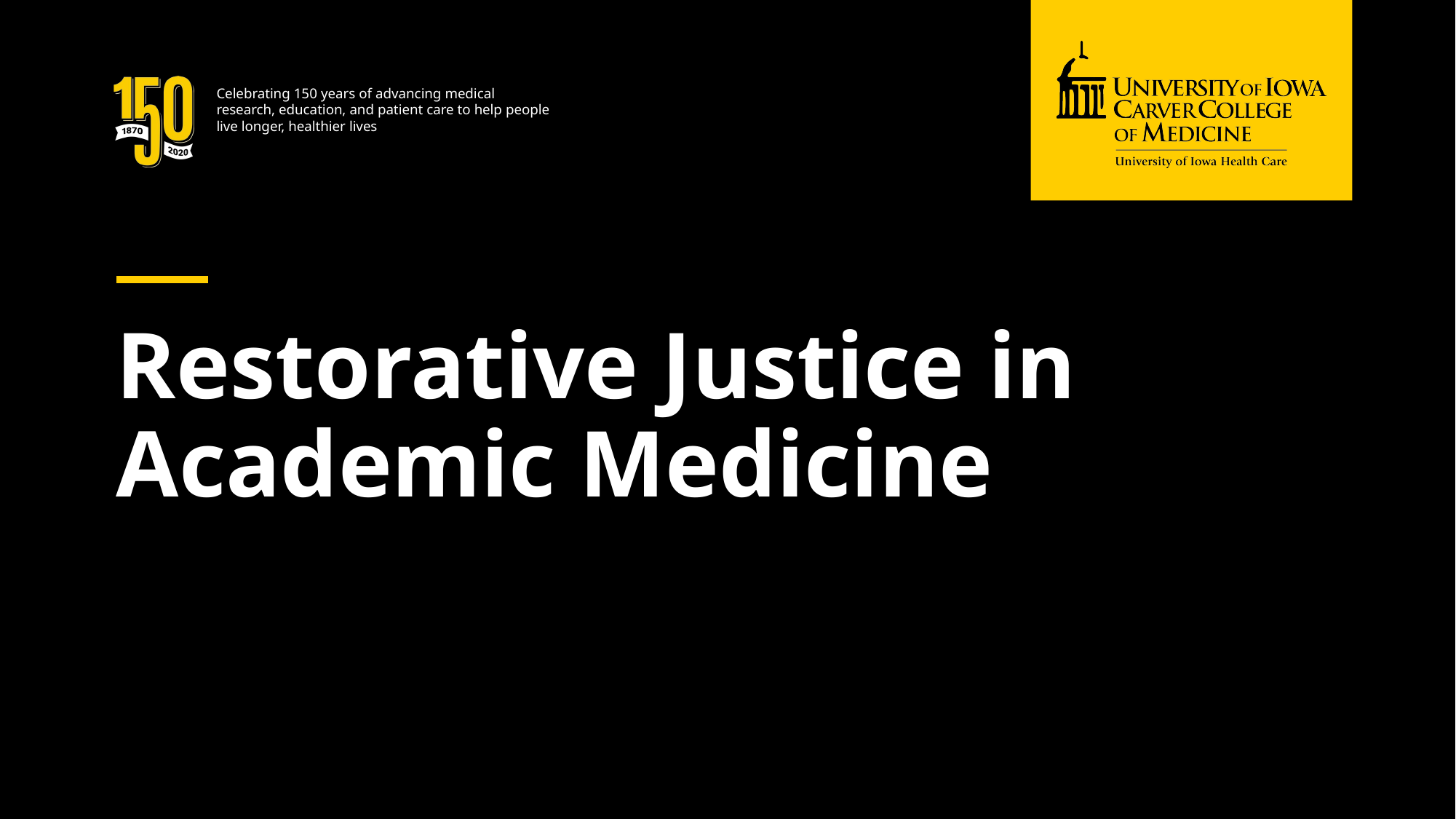

Celebrating 150 years of advancing medical research, education, and patient care to help people
live longer, healthier lives
# Restorative Justice in Academic Medicine

## Slide 2
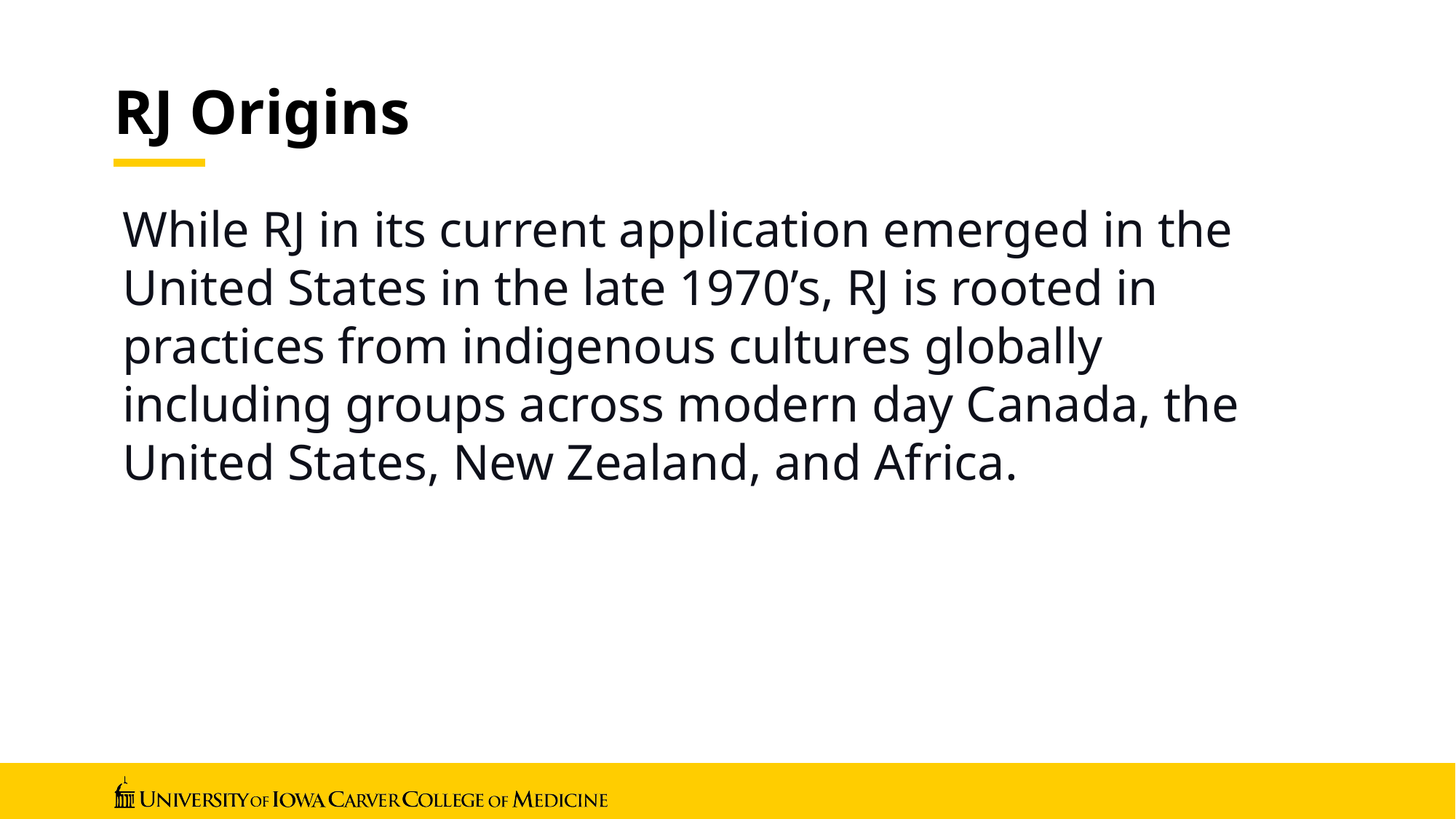

# RJ Origins
While RJ in its current application emerged in the United States in the late 1970’s, RJ is rooted in practices from indigenous cultures globally including groups across modern day Canada, the United States, New Zealand, and Africa.

## Slide 3
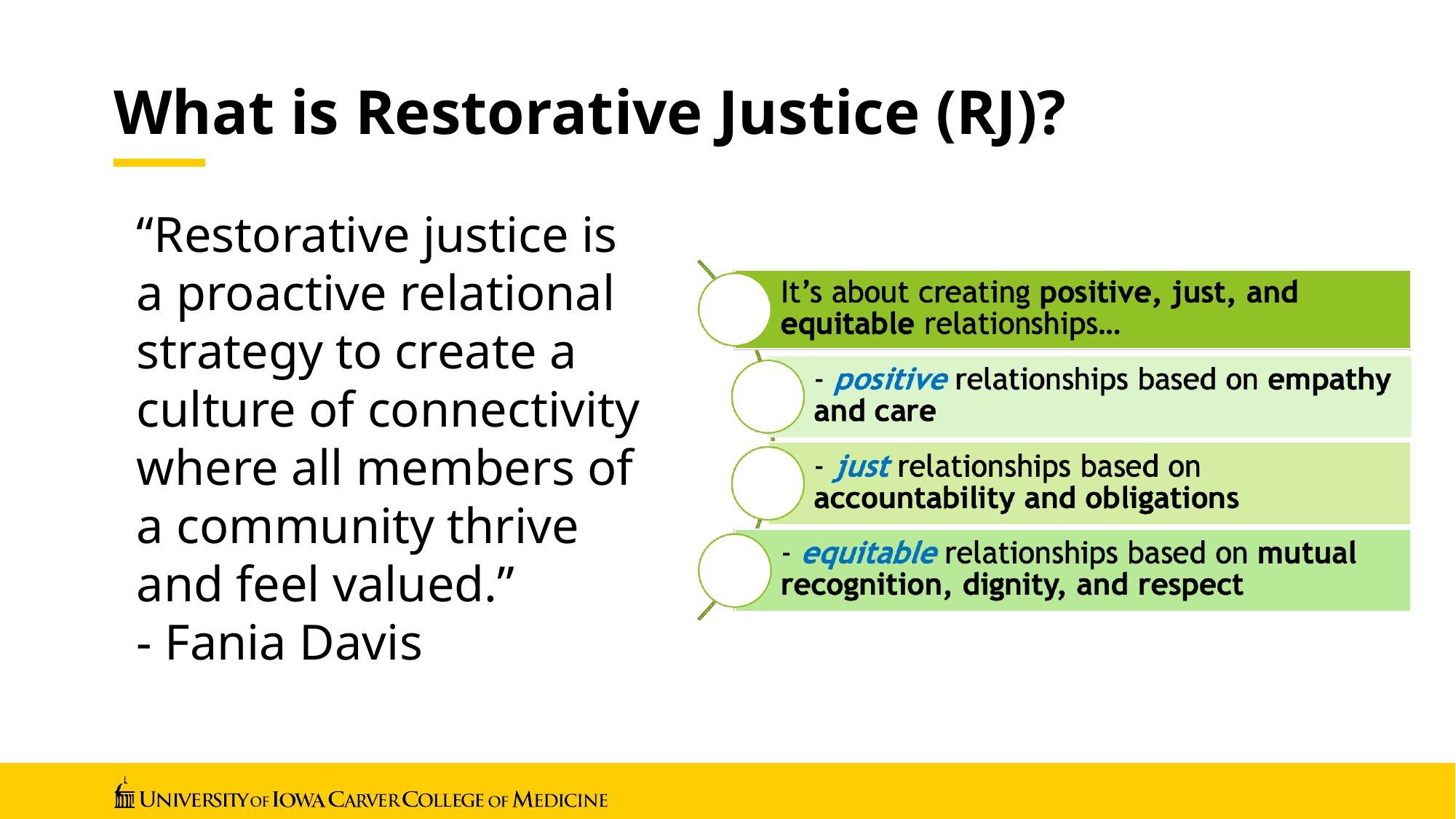

# What is Restorative Justice (RJ)?
“Restorative justice is a proactive relational strategy to create a culture of connectivity where all members of a community thrive and feel valued.”
- Fania Davis

## Slide 4
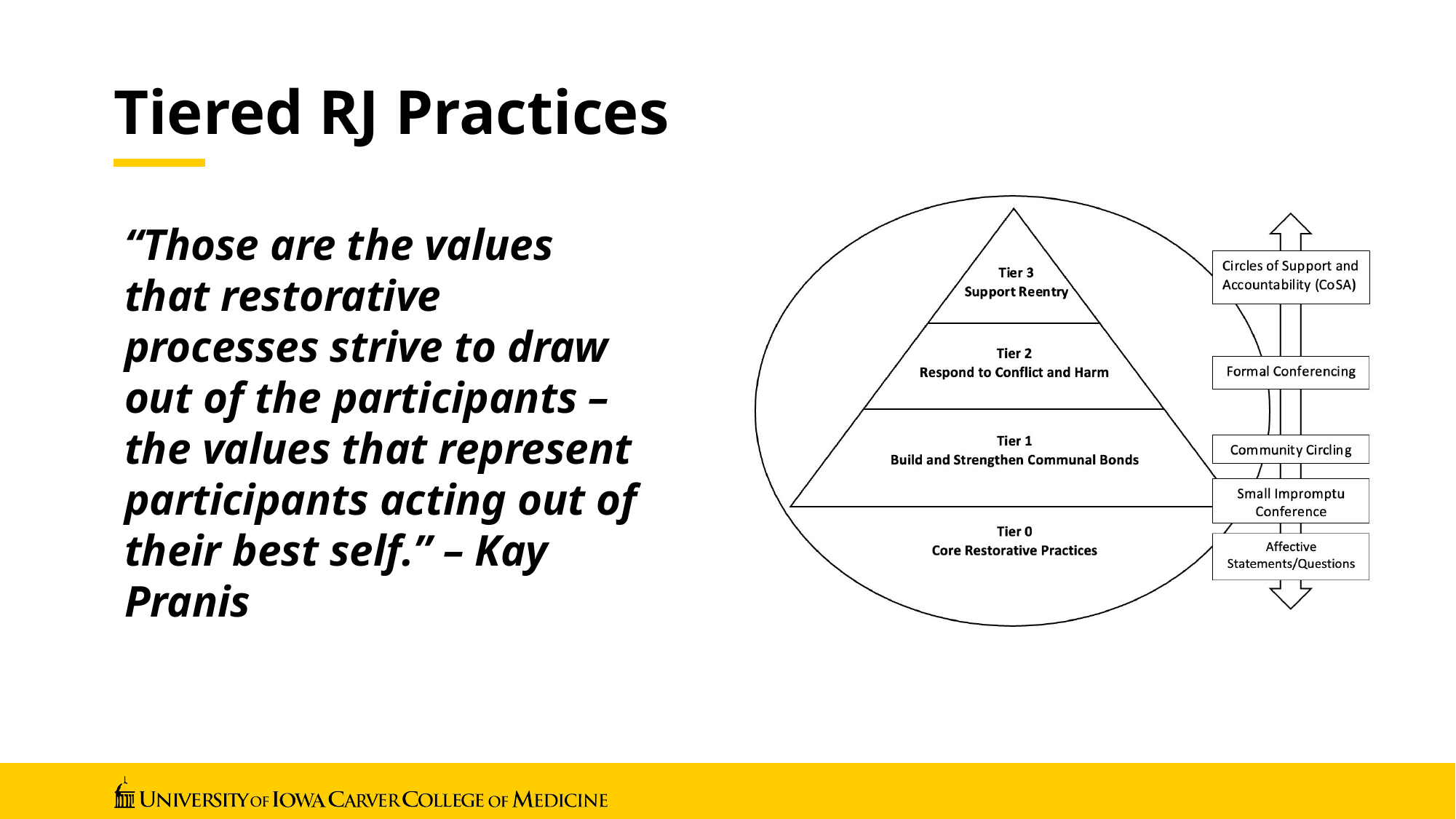

# Tiered RJ Practices
“Those are the values that restorative processes strive to draw out of the participants – the values that represent participants acting out of their best self.” – Kay Pranis

## Slide 5
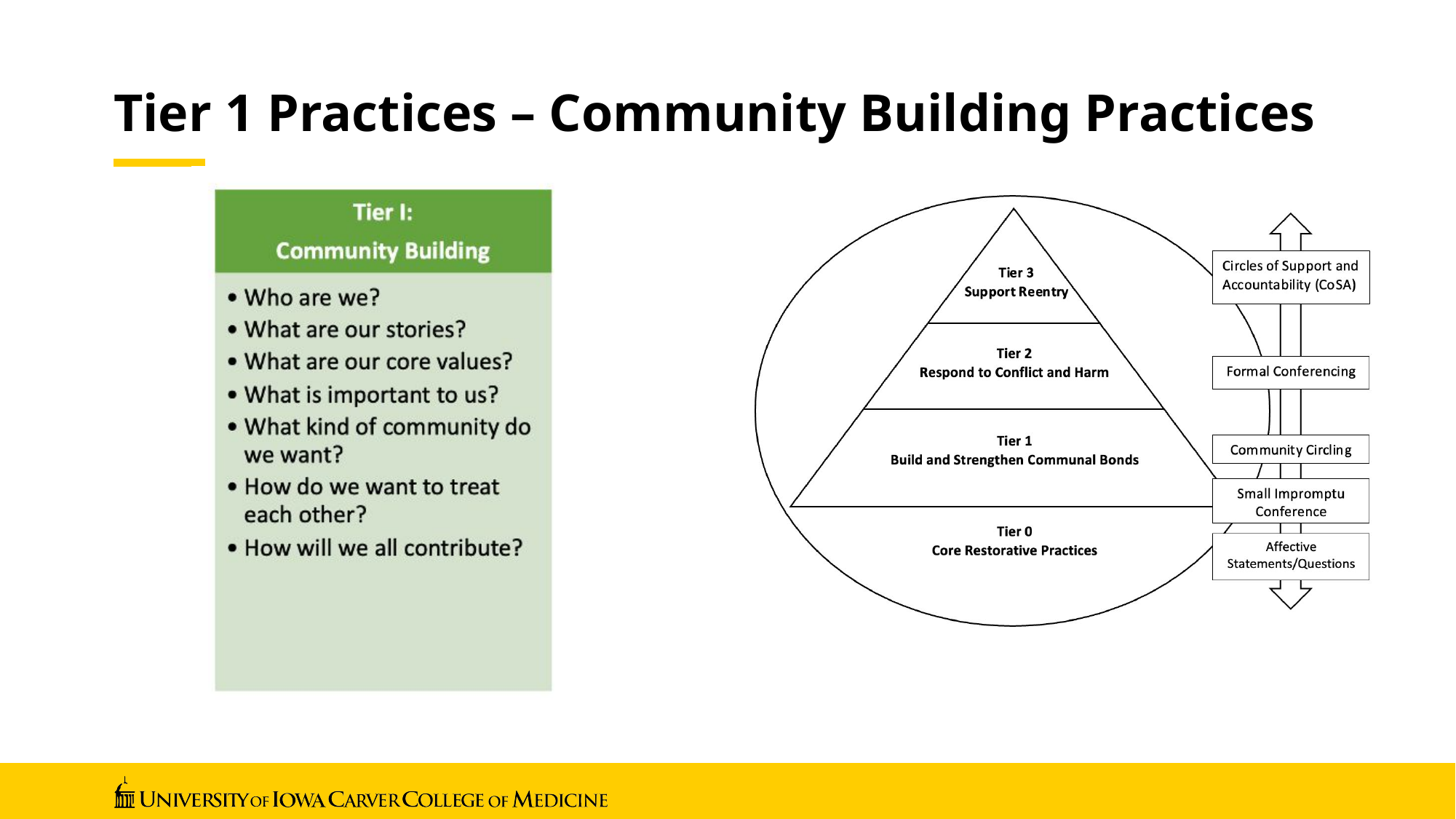

# Tier 1 Practices – Community Building Practices

## Slide 6
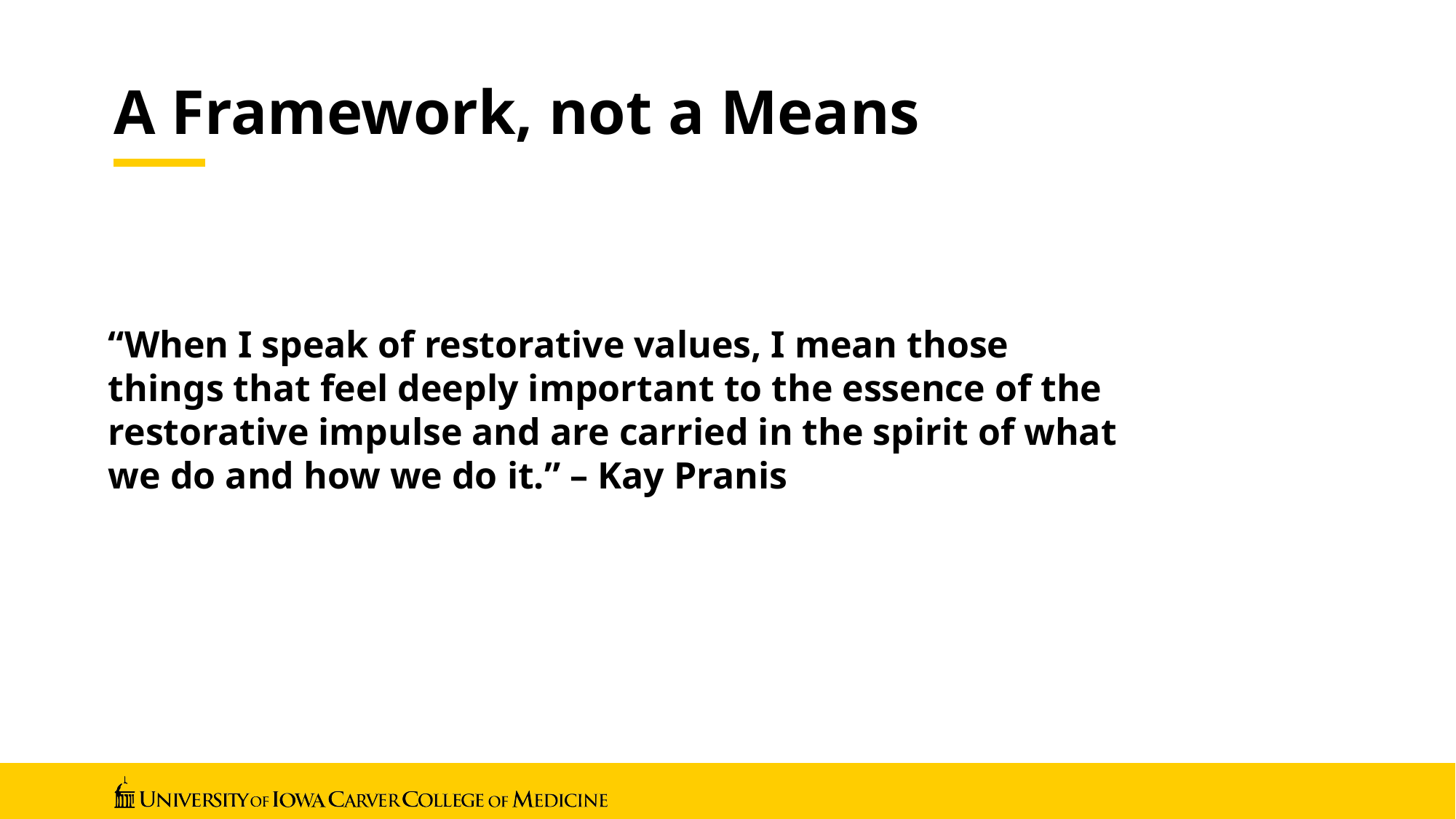

# A Framework, not a Means
“When I speak of restorative values, I mean those things that feel deeply important to the essence of the restorative impulse and are carried in the spirit of what we do and how we do it.” – Kay Pranis

## Slide 7
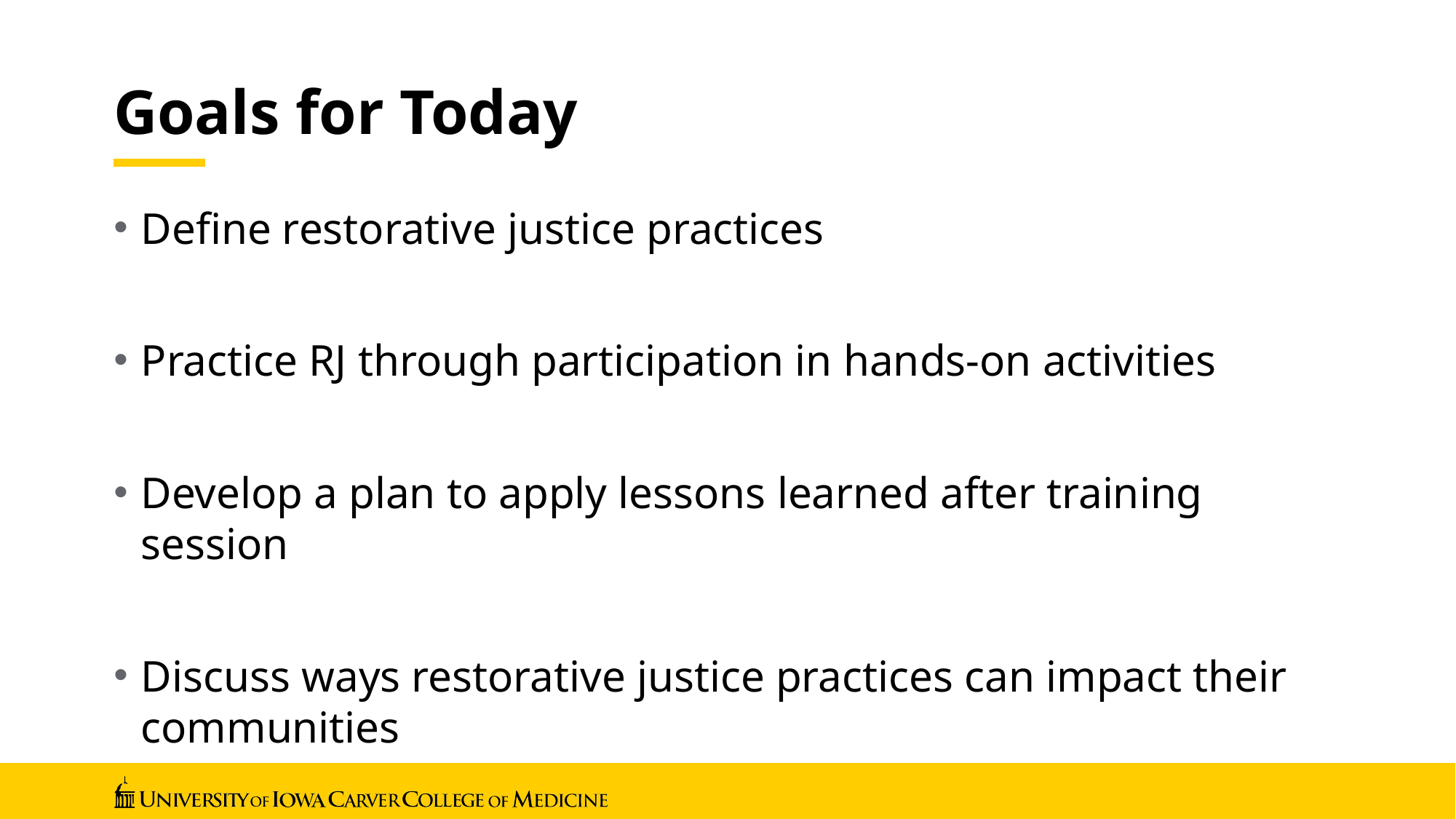

# Goals for Today
Define restorative justice practices
Practice RJ through participation in hands-on activities
Develop a plan to apply lessons learned after training session
Discuss ways restorative justice practices can impact their communities

## Slide 8
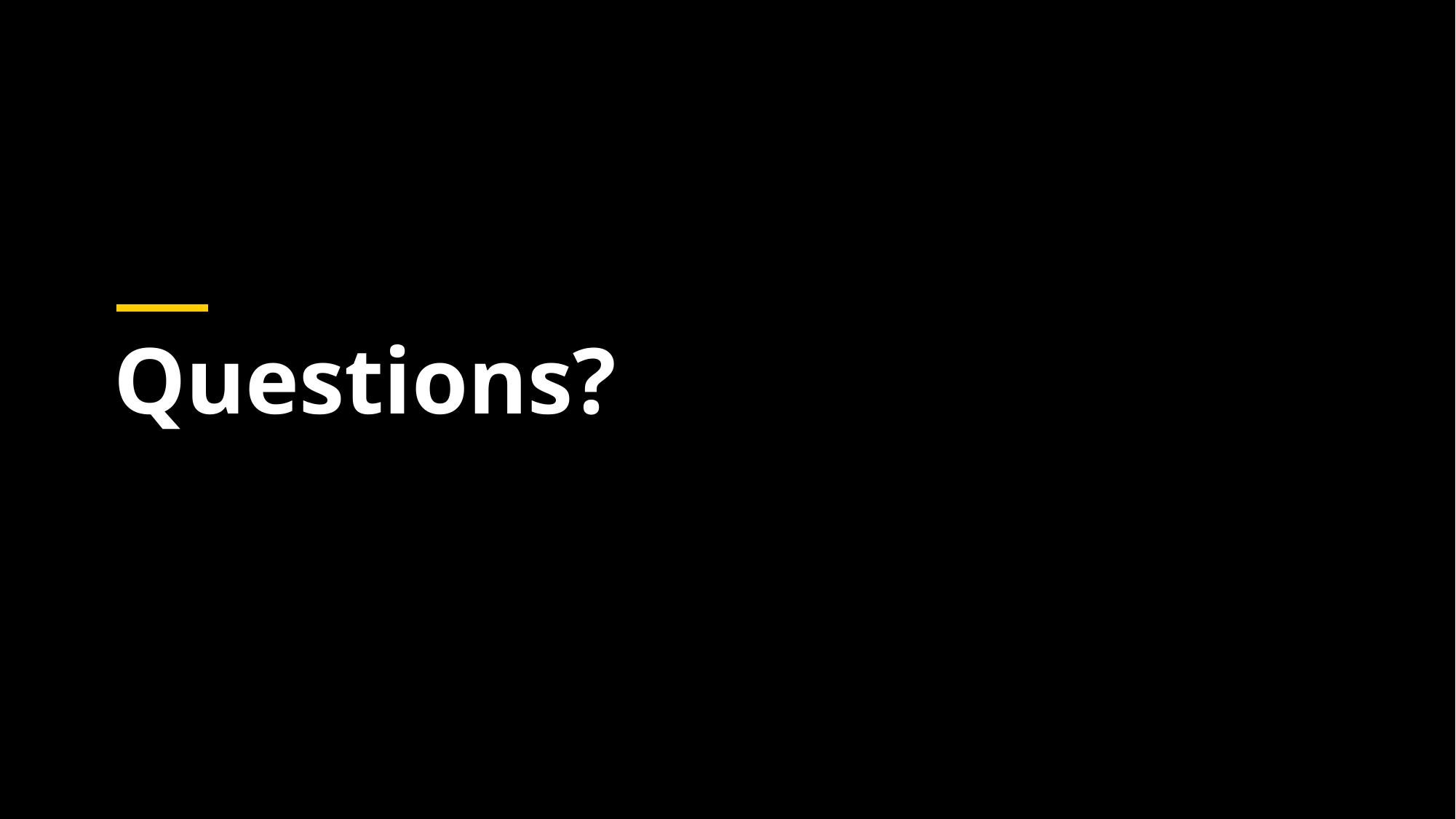

# Questions?
