# Supplementary material for: Restorative Community Building Practices: A Train-the-Trainer Workshop for Medical Students, Staff, and Faculty
Source: MedEdPORTAL. 2025 Sep 23;21:11547. doi: 10.15766/mep_2374-8265.11547 (PMC12454668; doi:10.15766/mep_2374-8265.11547)
Supplement: Supplementary file 1 — Training Schedule.docxRP Training Lecture 1.pptxRP Training Circle Scripts.docxRP in Academic Medicine.docxRP Training Lecture 2.pptxWorkshop Pre- and Postsurveys.docx3-Month Follow-Up Survey.docx [file mep_2374-8265.11547-s001.zip › C. RP Training Circle Scripts.docx]

Appendix C: RJ training circle scripts

Purpose: This appendix will be used by the circle keepers for the first activity of the workshop. This includes the specific script to be used by the keepers as well as key instructional points to include while working through the activity.

**Large Group Circle Activity:** This circling activity was performed to:

- Introduce participants to the circling process
- Establish knowledge of key circling concepts and rules.

This document both reflects the circling script questions (bolded print) as well as key teaching points that were emphasized at this time (italicized print).

Introduce Talking Piece **–**

The talking piece is:

- A *critical element in creating a space where everyone can both speak and listen*

*- A small object of significance that can be easily passed from person to person*

*- An everyday object can be used, but the keeper should explain how it connects and its meaning in this space.*

- **Person with talking piece speaks without interruption, participants listen without a need to respond.**
- **Passed around from person to person always in a circle unless the keeper suspends the talking piece for a popcorn response or some other reason.**
- **You may always pass if you do not wish to share anything.**
- **Keeper can speak without piece if needed to address questions and the like.**

**We’ll practice the use of the talking piece with the Check-in Round. This round is meant to warm-up participants, to connect them, to prepare them for deeper conversation that will be built by talking about the small stuff first.**

**It’s often helpful to start with a round to learn names when in new groups—the foundation of relationship is knowing each other, who we are, and that often starts with how we are called.**

*Notes:*

*-For the above paragraphs, you don’t have to introduce these concepts verbatim, but we believe they are helpful ways to orient towards the activity.*

1- Check-in Round -

**Prompt: Share your name with a word that starts with the same letter that describes something about you. After stating your name and adjective**, **recite the adjectives and names of the people prior to them.**

*Keeper models response, e.g. Amazon Ada, Mango Maya. The next person will introduce their adjective and name.* They cannot pass until they have gotten everyone’s name.

Note:

-If someone cannot remember a name, the group is encouraged to help and provide that information. This activity is meant to be a fun opener, not a test.

- Note taking to remember names, or otherwise, is discouraged as circling rules usually ask for no phones, notes, etc. unless otherwise specified to encourage participants to be present and focus on listening. This is why the group is allowed to help each other through this activity.

2- Values Round -

*RJ is rooted in values (rather than rules) and part of the practice is to proactively agree to how we show up and how we engage with each other.*

*Establishing norms cues behavior which makes it more likely that we will abide by the values, expectations or guidelines that we set:*

*- It allows individuals to share what they need from their lived experience (rather than assuming we all need the same things);*

*- It allows us to clarify what we mean by certain values:*

*- How we enact or practice them.*

*- What’s needed to create a more equitable space? Specifically, what values or ground rules do we need?*

**Prompt: What values do you need from the group today?** *Keeper models brief response.*

After the group sets its own and if they aren’t already raised by the group:

**In addition to keeping these values in mind, we’ll also want to:**

- **Listen to understand, not to respond. So should share from your own lived experience (I statements, not you statements.)**
- **Speak and listen from the heart: assume good intent; acknowledge multiple truths.**
- **Stories stay, lessons leave: confidentiality.**
- **Offer your full presence:no phones.**

*Things to establish as a keeper for the group: Listening can be exhausting – we did this together in part to recognize what it requires to truly create space for everyone. We often feel like we don’t have time for such things. Yet, we spend a lot of time working through problems that might have been avoided had we created more understanding between ourselves – this kind of prevention is at the heart of restorative practices.*

*Recognizing that we can’t do this always, we’ll be modeling how you can also use these practices in a mix of ways.*

*For the next activity, we’re going to break up into smaller circles for the next rounds. Each of you were asked to bring an object of significance and these next rounds will provide you an opportunity to share these stories with your colleagues.*

**Small Group Circle Activity:**

- This portion of the circle was completed in smaller groups immediately after finishing the values round.
- In totality, the large and small group activities are a complete circle script that was partially completed in a large group setting to communicate key concepts and for the workshop attendees to familiarize themselves with each other.
- Like the previous section, circle prompts are in bold print, and teaching points are in italicized print.
- For this activity, participants will need to have brought an item of personal importance, which requires communication prior to the workshop. Participants do not need to bring anything else.

1^st^ Round:

**Prompt: Share what you’ve brought, the story of what it means to you in about 3-4 minutes and place it in the center of the circle.**

- *Keeper models a 3-4 minute story. The level of vulnerability that the keeper models will set the tone for the circle.*

*-In general, in circling, the facilitator has a key role in modeling, or setting the example for, the type and length of response encouraged.*

*- The talking piece does not need to be used for this round as placing one’s object in the middle will signify “passing” the talking piece.*

2^nd^ Round:

**Prompt: For this round, pick someone else’s object--an object whose significance resonated with you in some way. Each object may only be picked one time. Share with the group what you heard about the object, how that resonates to a story from your life and then return it to the owner**.

- *Keeper models picking an object, summarizing the object owner’s story, sharing a 2-minute story from their life that connects to those thoughts, and then returns the object to the owner.*

*- Similar to the last round, no talking piece is needed and returning the object back to its original owner will signify “passing” the talking piece.*

Check-out round:

**Prompt:** **Share something that you are taking from the circle this morning.**

*- The talking piece will be used again for this round.*

Closing:

*- Keeper thanks everyone for sharing and may offer a reflection of themes or connections.*

**Prompt: Just like we open the circle to signify the start of a different kind of space, we also typically close the circle to signify the ending – can be a quote, song, game, or some other ritual - For this circle, we’ll take three deep breaths together as we leave the space.**
