# Supplementary material for: Restorative Community Building Practices: A Train-the-Trainer Workshop for Medical Students, Staff, and Faculty
Source: MedEdPORTAL. 2025 Sep 23;21:11547. doi: 10.15766/mep_2374-8265.11547 (PMC12454668; doi:10.15766/mep_2374-8265.11547)
Supplement: Supplementary file 1 — Training Schedule.docxRP Training Lecture 1.pptxRP Training Circle Scripts.docxRP in Academic Medicine.docxRP Training Lecture 2.pptxWorkshop Pre- and Postsurveys.docx3-Month Follow-Up Survey.docx [file mep_2374-8265.11547-s001.zip › E. RP Training Lecture 2.pptx]

## Slide 1
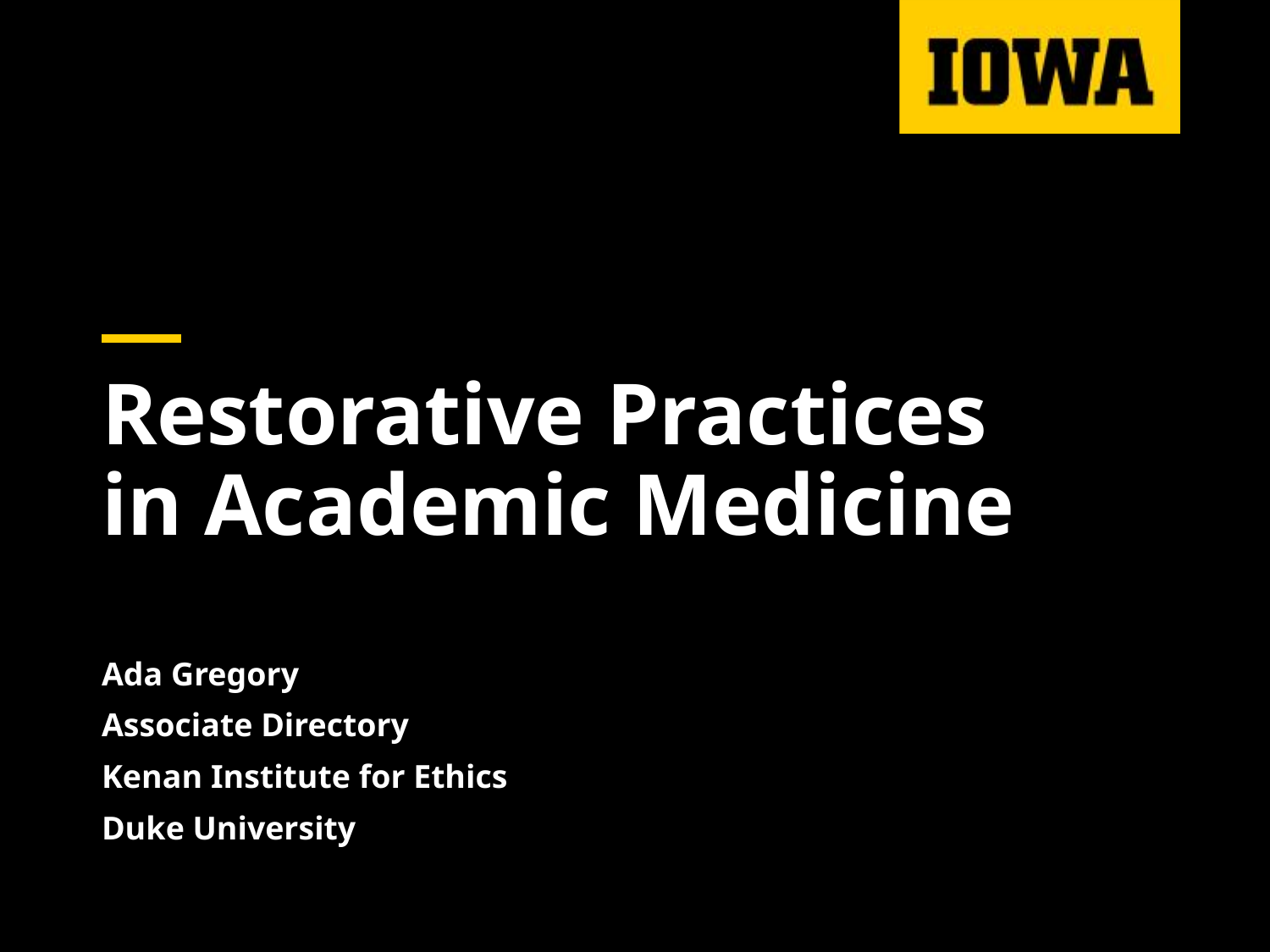

# Restorative Practices in Academic Medicine
Ada Gregory
Associate Directory
Kenan Institute for Ethics
Duke University

## Slide 2
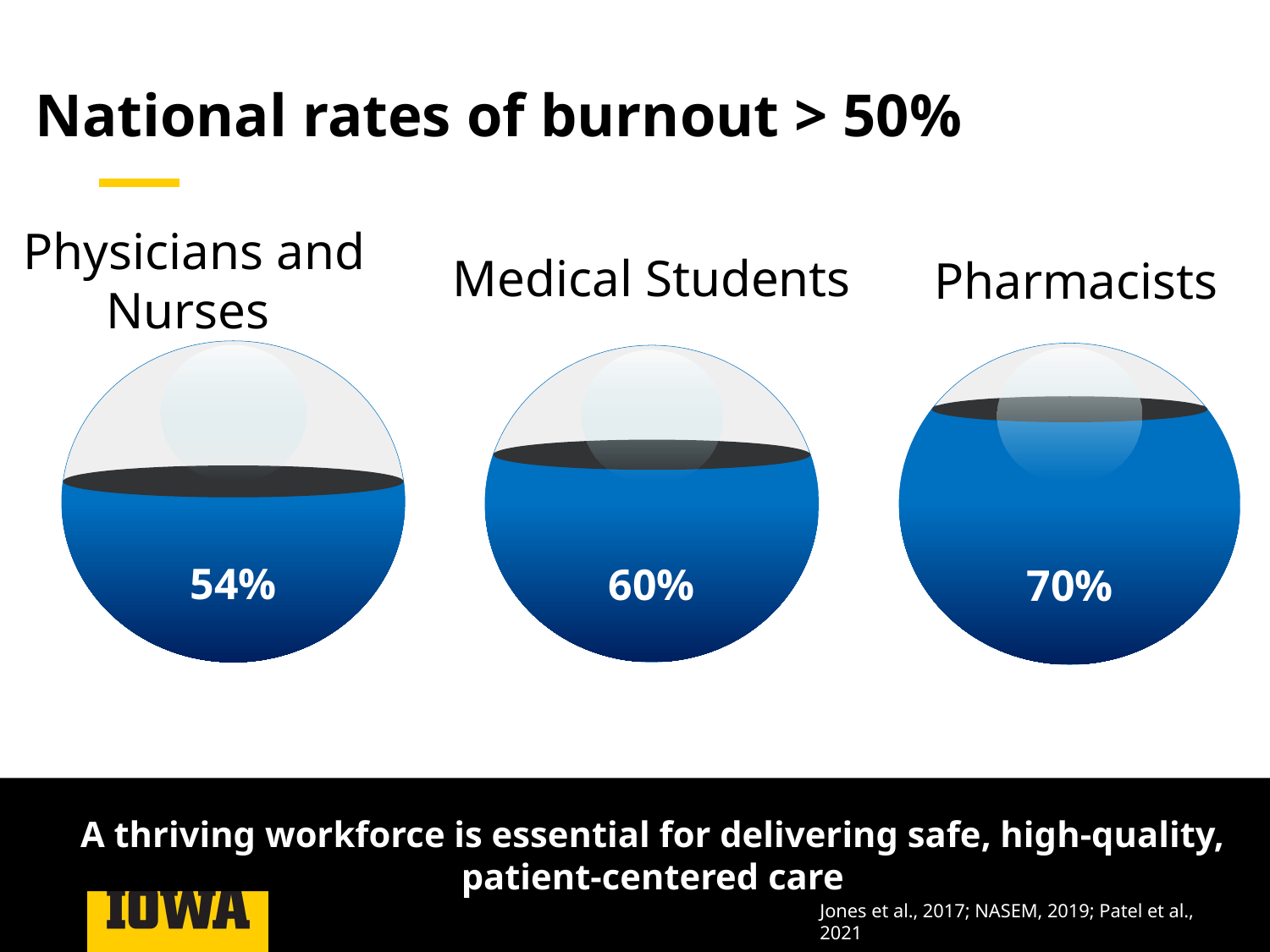

# National rates of burnout > 50%
Medical Students
Physicians and Nurses
Pharmacists
54%
70%
60%
75%
50%
A thriving workforce is essential for delivering safe, high-quality, patient-centered care
Jones et al., 2017; NASEM, 2019; Patel et al., 2021

## Slide 3
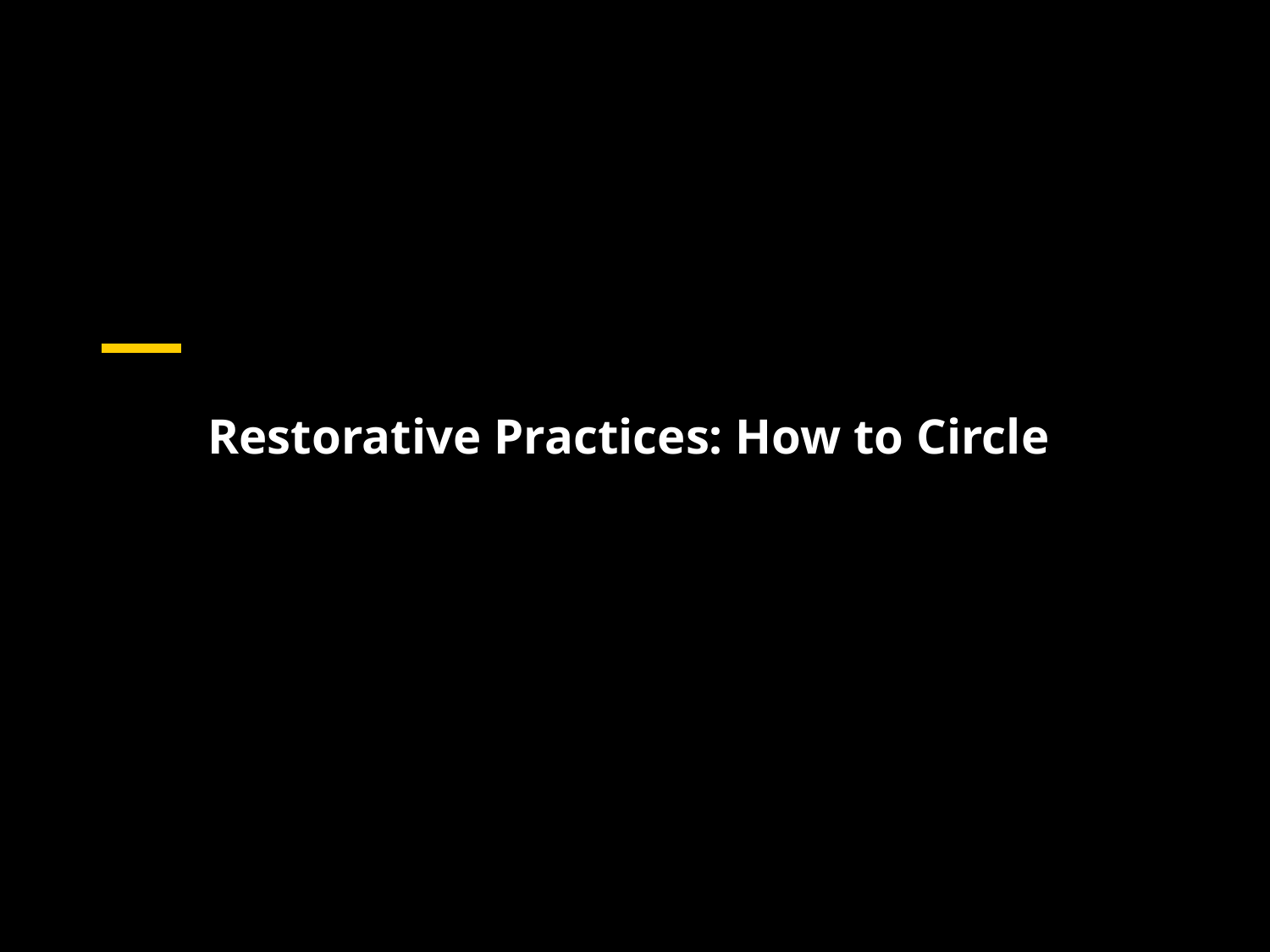

# Restorative Practices: How to Circle

## Slide 4
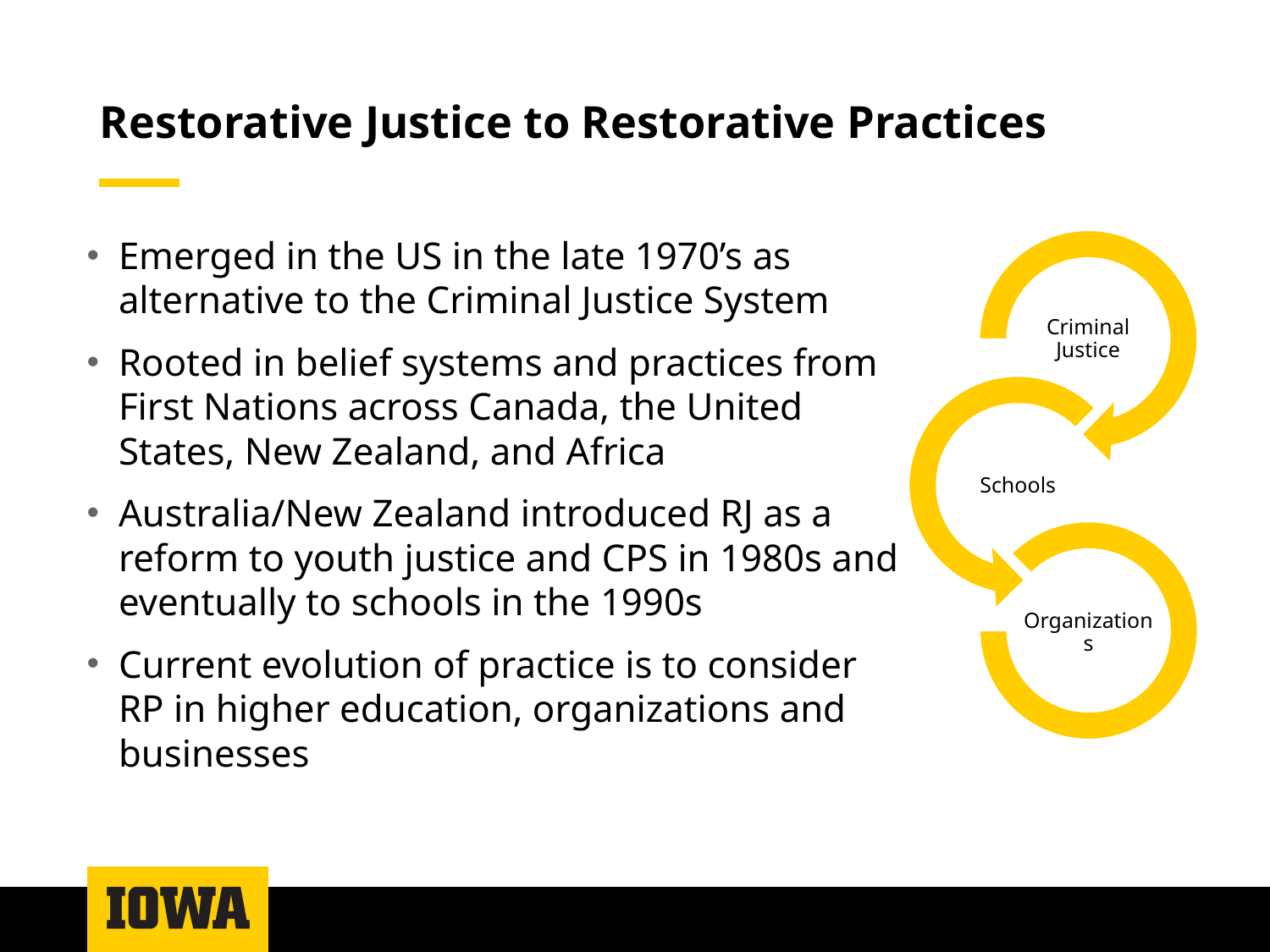

# Restorative Justice to Restorative Practices
Emerged in the US in the late 1970’s as alternative to the Criminal Justice System
Rooted in belief systems and practices from First Nations across Canada, the United States, New Zealand, and Africa
Australia/New Zealand introduced RJ as a reform to youth justice and CPS in 1980s and eventually to schools in the 1990s
Current evolution of practice is to consider RP in higher education, organizations and businesses

## Slide 5
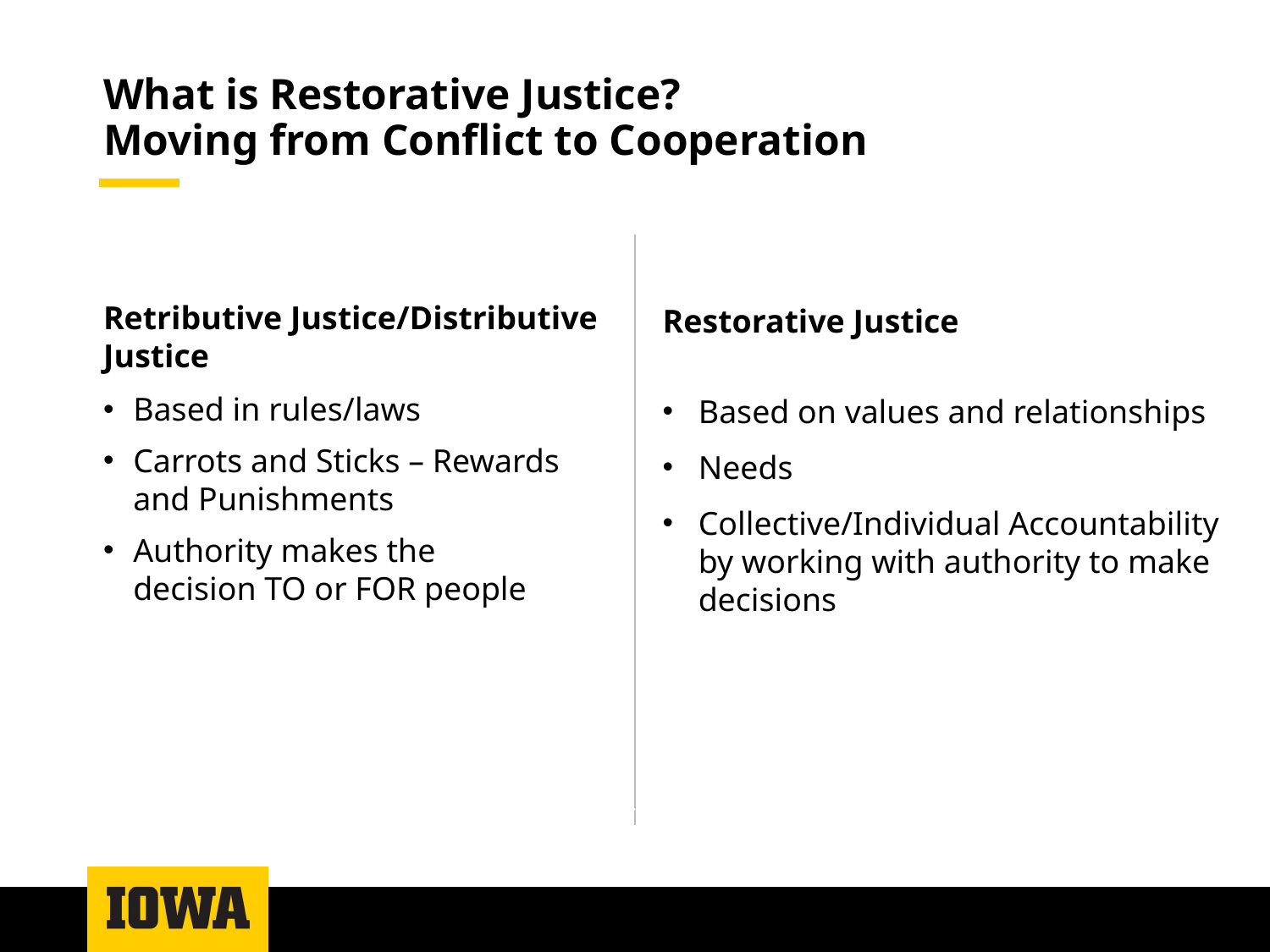

# What is Restorative Justice? Moving from Conflict to Cooperation
Restorative Justice
Retributive Justice/Distributive Justice
Based in rules/laws
Carrots and Sticks – Rewards and Punishments
Authority makes the decision TO or FOR people
Based on values and relationships
Needs
Collective/Individual Accountability by working with authority to make decisions
View >> Header and Footer >> Add Unit Name

## Slide 6
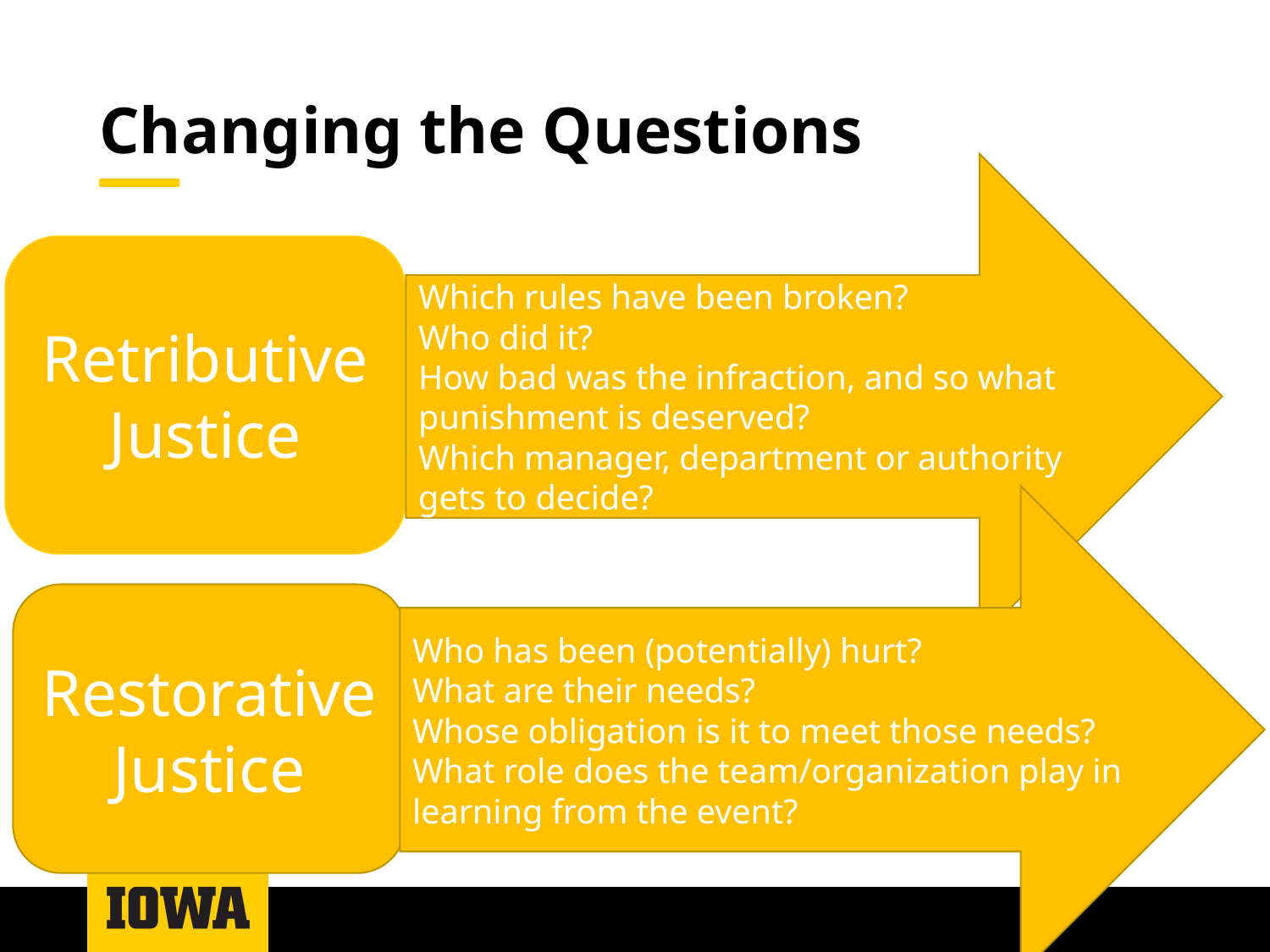

# Changing the Questions
Which rules have been broken?
Who did it?
How bad was the infraction, and so what punishment is deserved?
Which manager, department or authority gets to decide?
Retributive Justice
Who has been (potentially) hurt?
What are their needs?
Whose obligation is it to meet those needs?
What role does the team/organization play in learning from the event?
75%
Restorative Justice
50%

## Slide 7
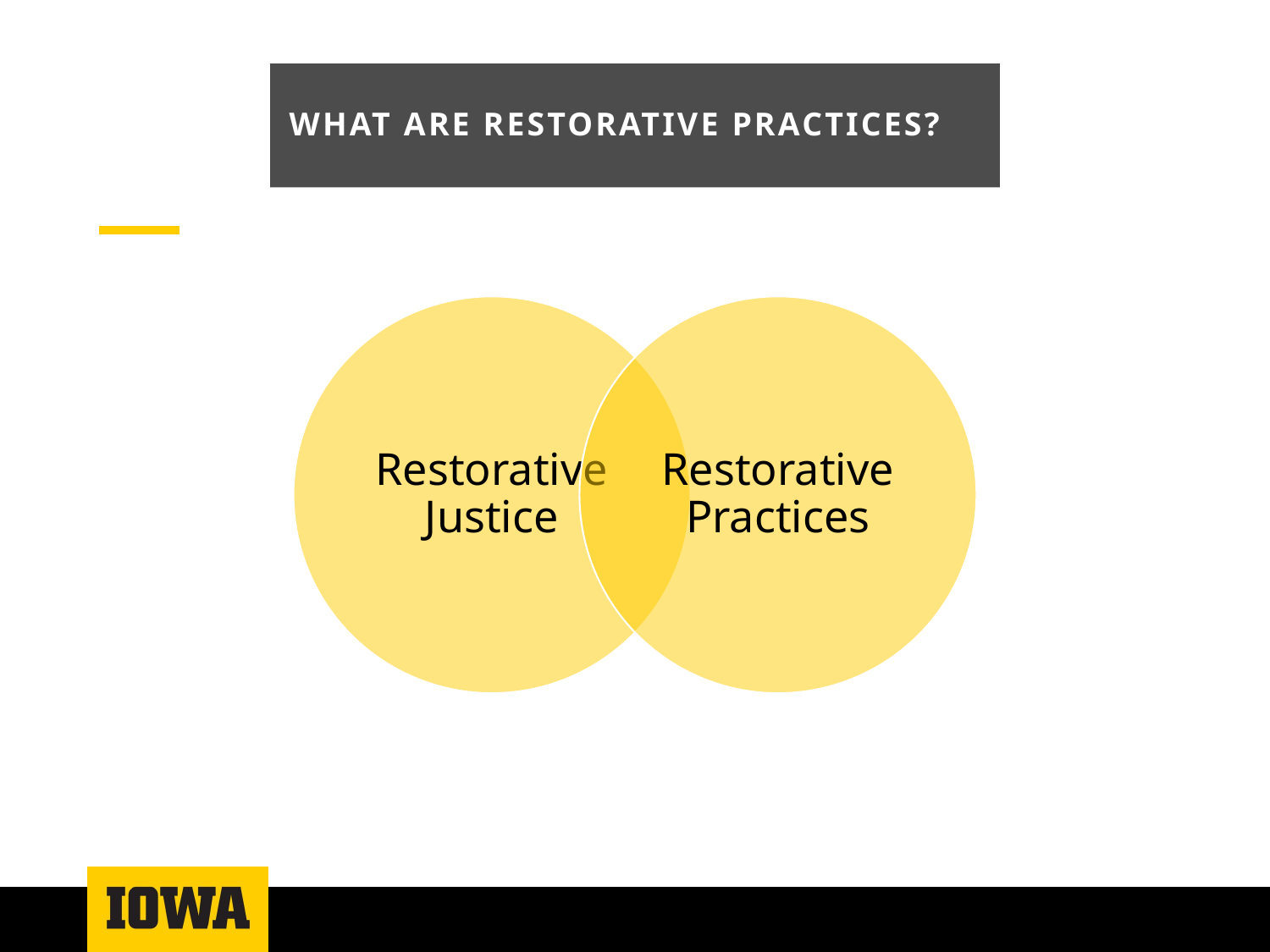

# What Are Restorative Practices?

## Slide 8
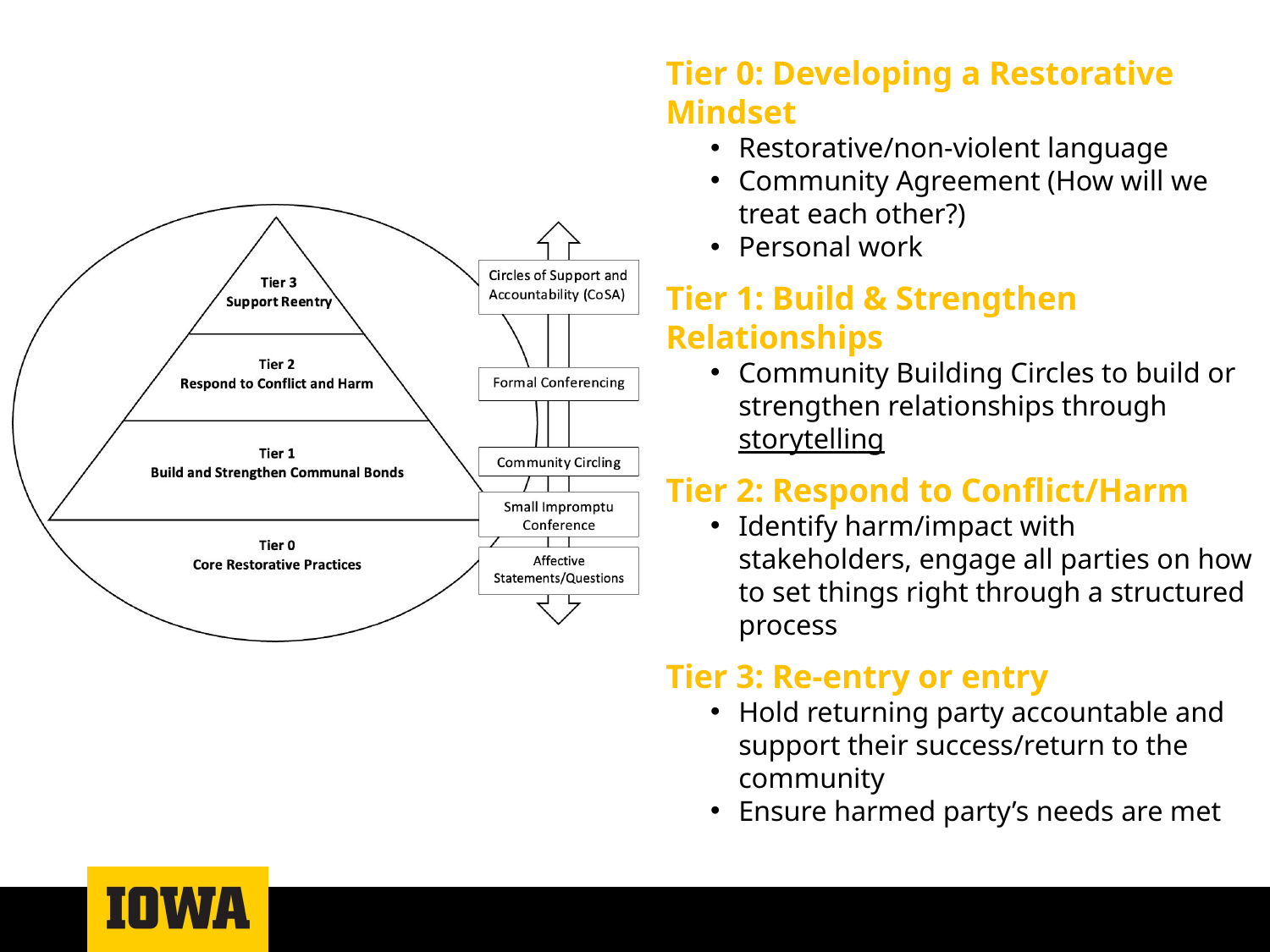

Tier 0: Developing a Restorative Mindset
Restorative/non-violent language
Community Agreement (How will we treat each other?)
Personal work
Tier 1: Build & Strengthen Relationships
Community Building Circles to build or strengthen relationships through storytelling
Tier 2: Respond to Conflict/Harm
Identify harm/impact with stakeholders, engage all parties on how to set things right through a structured process
Tier 3: Re-entry or entry
Hold returning party accountable and support their success/return to the community
Ensure harmed party’s needs are met
Sawin et al, 2023

## Slide 9
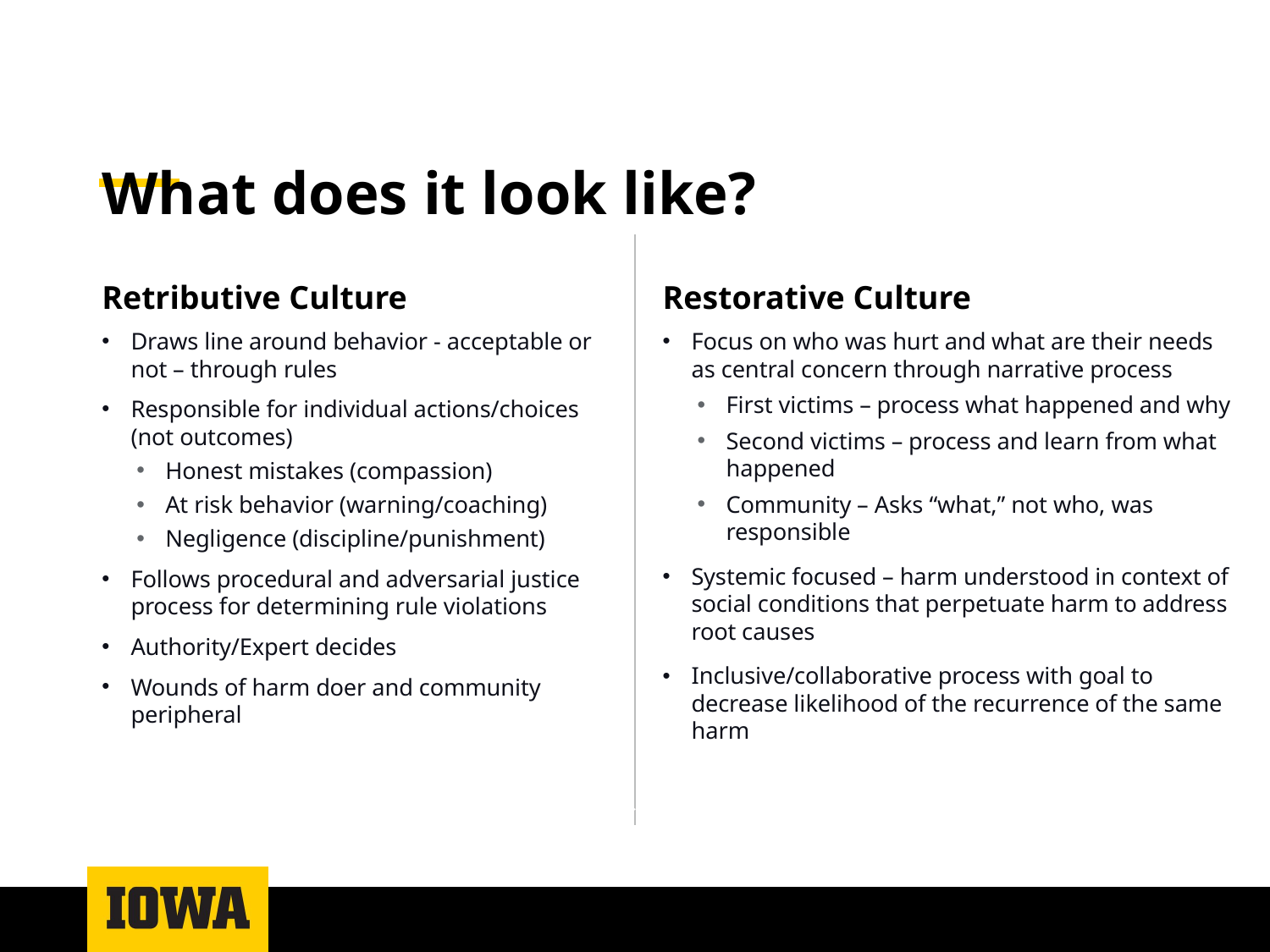

# What does it look like?
Retributive Culture
Restorative Culture
Draws line around behavior - acceptable or not – through rules
Responsible for individual actions/choices (not outcomes)
Honest mistakes (compassion)
At risk behavior (warning/coaching)
Negligence (discipline/punishment)
Follows procedural and adversarial justice process for determining rule violations
Authority/Expert decides
Wounds of harm doer and community peripheral
Focus on who was hurt and what are their needs as central concern through narrative process
First victims – process what happened and why
Second victims – process and learn from what happened
Community – Asks “what,” not who, was responsible
Systemic focused – harm understood in context of social conditions that perpetuate harm to address root causes
Inclusive/collaborative process with goal to decrease likelihood of the recurrence of the same harm
View >> Header and Footer >> Add Unit Name

## Slide 10
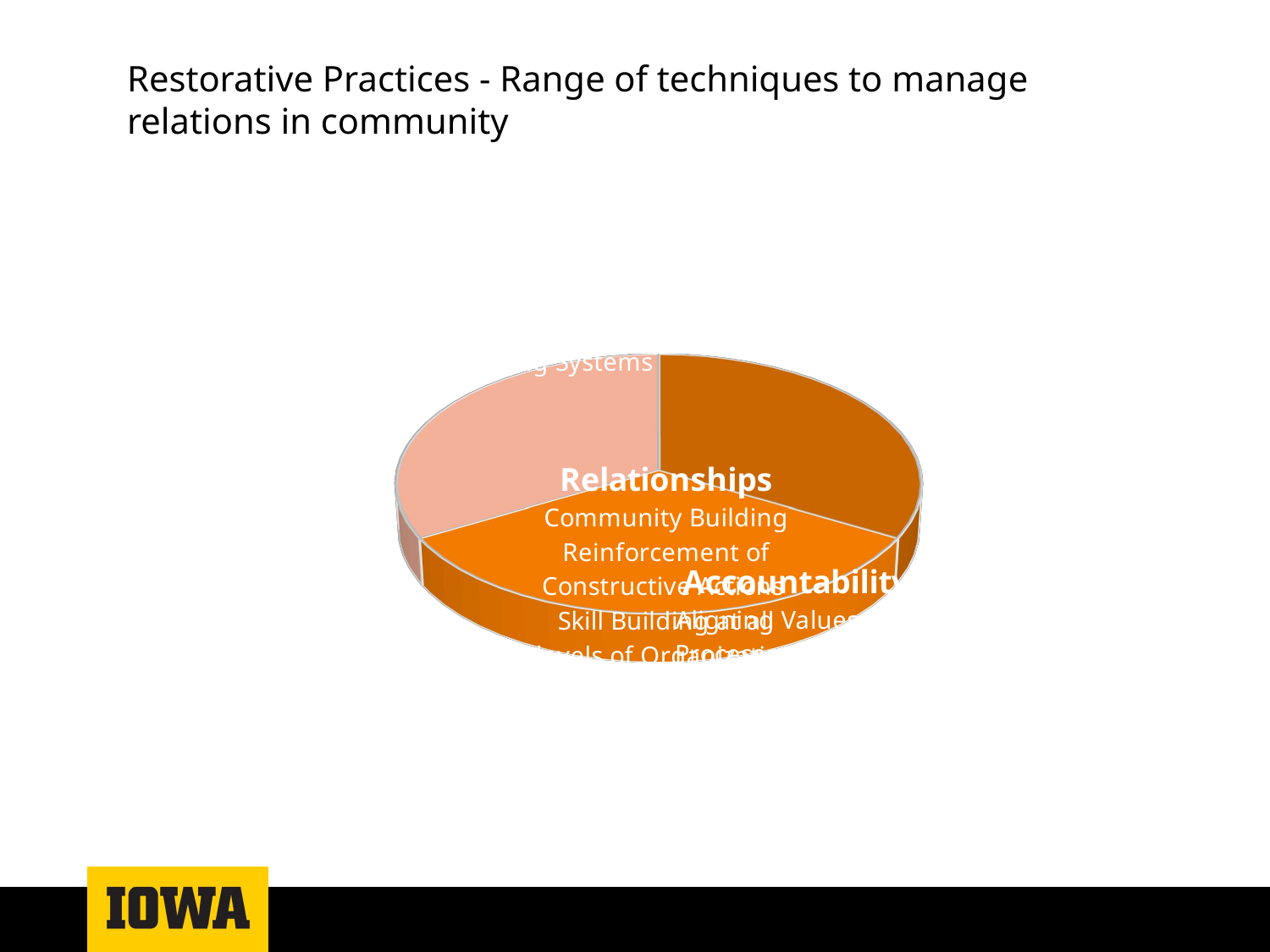

Restorative Practices - Range of techniques to manage relations in community
[unsupported chart]

## Slide 11
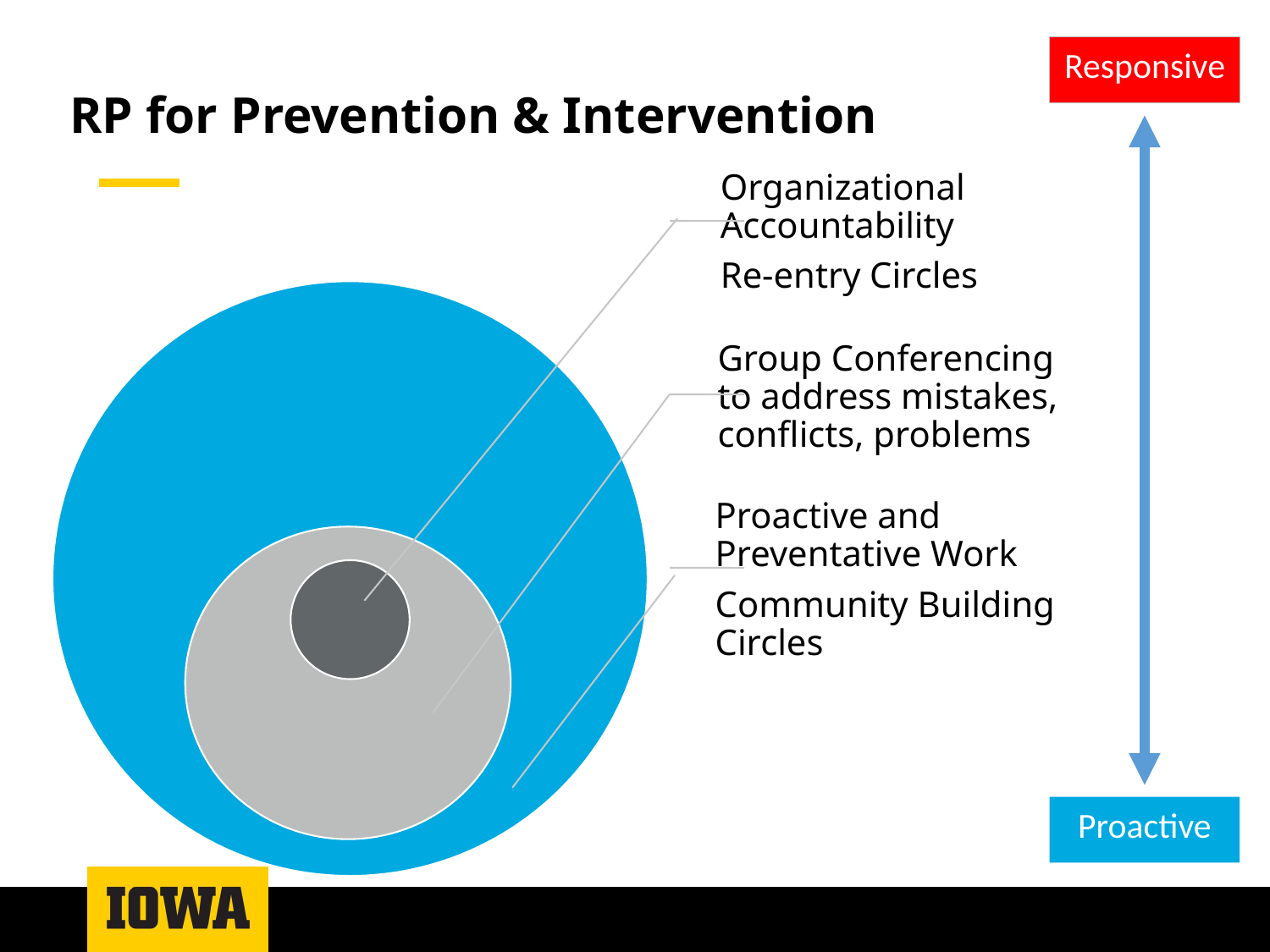

Responsive
RP for Prevention & Intervention
Proactive

## Slide 12
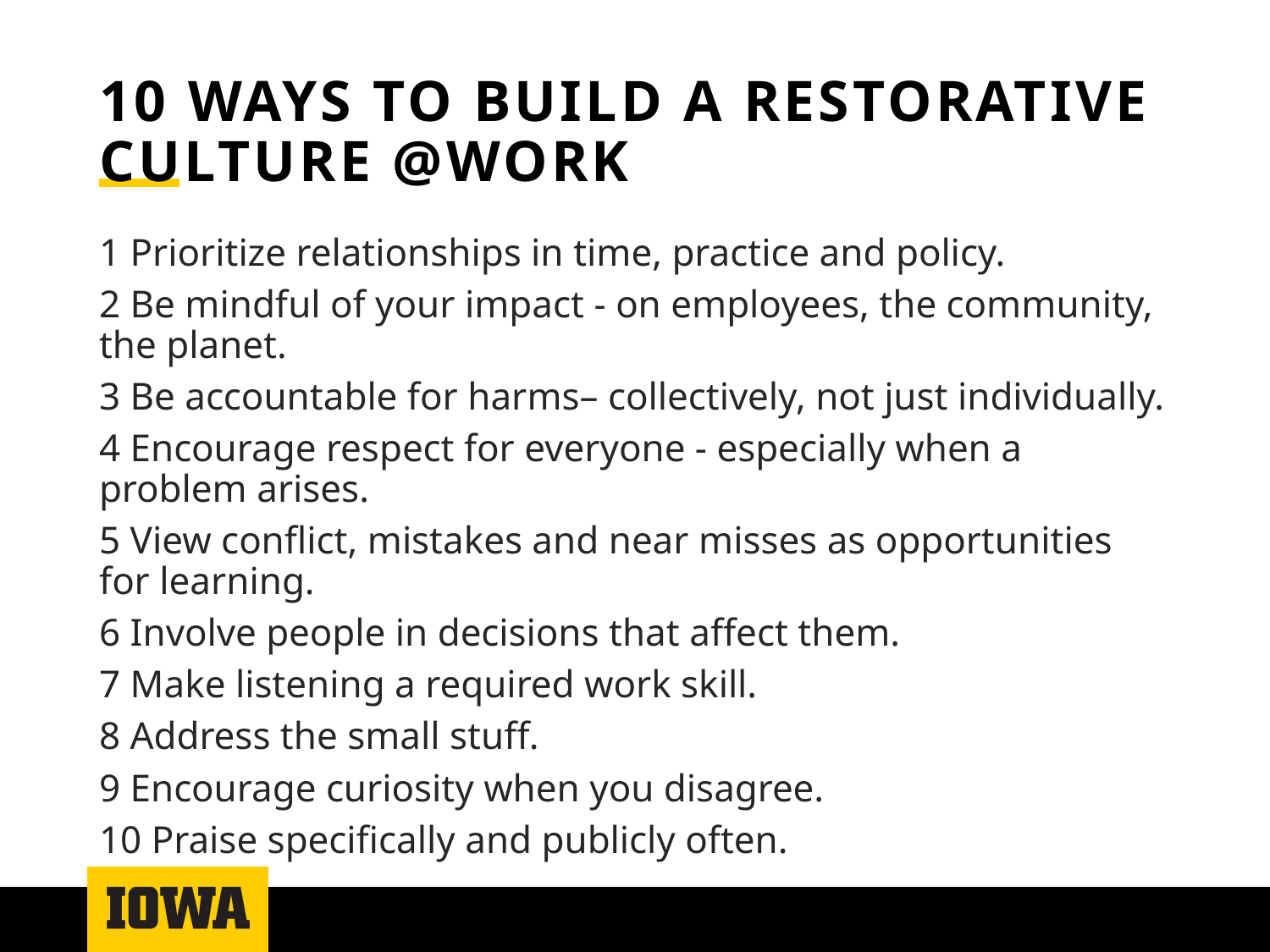

# 10 ways to build a restorative culture @work
1 Prioritize relationships in time, practice and policy.
2 Be mindful of your impact - on employees, the community, the planet.
3 Be accountable for harms– collectively, not just individually.
4 Encourage respect for everyone - especially when a problem arises.
5 View conflict, mistakes and near misses as opportunities for learning.
6 Involve people in decisions that affect them.
7 Make listening a required work skill.
8 Address the small stuff.
9 Encourage curiosity when you disagree.
10 Praise specifically and publicly often.
