# Supplementary material for: Restorative Community Building Practices: A Train-the-Trainer Workshop for Medical Students, Staff, and Faculty
Source: MedEdPORTAL. 2025 Sep 23;21:11547. doi: 10.15766/mep_2374-8265.11547 (PMC12454668; doi:10.15766/mep_2374-8265.11547)
Supplement: Supplementary file 1 — Training Schedule.docxRP Training Lecture 1.pptxRP Training Circle Scripts.docxRP in Academic Medicine.docxRP Training Lecture 2.pptxWorkshop Pre- and Postsurveys.docx3-Month Follow-Up Survey.docx [file mep_2374-8265.11547-s001.zip › D. RP in Academic Medicine.docx]

## **Appendix D: RP in Academic Medicine**

## Purpose: To provide a worksheet with a framework for utilizing affective statements and questions as well as designing a circle. This will be utilized for the portion of the training where participants design and practice their own circles.

## **Community Building Circle Exercise**

***PART I Planning***

Who is the circle for? How will they be invited to participate?

For what purpose? What are your goals?

How much time do you have?

Where will you have it so you can ensure privacy and that all participants can sit in a circle without obstructions?

## **Affective Statements**

*Based on principles of Non-Violent Communication from Rosenberg MB, Chopra D. Nonviolent Communication: A Language of Life: Life-Changing Tools for Healthy Relationships. PuddleDancer Press; 2015.

Feedback to reinforce constructive behaviors and discussion to redirect unconstructive ones, moving from behavior management to *relationship* management

**Observation:** What happened - When you____________

**Feeling**s: How it affected you or others – words that describe emotions

**Needs**: Associate needs/values behind the feeling - because _________________.

**Behavior Request:** What would meet the needs?

| **Typical Response** | **Affective Statement** |
| --- | --- |
| Thanks for helping out! | *When you jumped in to take care of that patient before your service even started, the whole team’s morale shifted for the better because they saw the dedication you have not only to the work but to them too.* |

For unconstructive, ***private*** *discussions* in a similar vein can be employed.

| **Typical Response** | **Affective Statement** |
| --- | --- |
| You are never on time. | *When you were late this morning, I worried about the team working overtime because we try to respect time off as much as worktime. Can we do something to address what’s getting in the way for you?* |

**Affective Questions Exercise**

*Adapted from Schein EH, Schein PA. Humble Inquiry, Second Edition: The Gentle Art of Asking Instead of Telling. Berrett-Koehler Publishers; 2021.

| **Situation** | **Typical Response** | **Affective Question** |
| --- | --- | --- |
| In a faculty meeting, a colleague is misrepresenting your work to cast her work in a more favorable light. | I don’t think that’s right. Your summary isn’t accurate. Where did you get your information? |  |
| A research team is underperforming due to unexplained delays or problems. You need to know what is going on, but you are not sure the team will be forthcoming. | All our jobs are on the line if we don’t make this grant project go without more delays. We need to get back on track. |  |
| Committee members are becoming noticeably less engaged in a major Schoolwide initiative. You are at a meeting in which senior leadership is not present. | I think we’re not working hard enough here. Let’s get with it. |  |
| You have a new assignment for a staff member, but you are not sure they will enthusiastically buy into this assignment, even if role may be a promotion. | I am going to assign you to support this new initiative. It’s a highly visible move, a promotion really. I hope you are as thrilled as I am. |  |

## **Community Building Circle Exercise**

***Part II Structure***

***Welcome & Opening*:** Welcome everyone to the circle and thank them for coming. Openings set the tone and mark the space as apart from the everyday work pace. It helps situate participants for what’s to come, remind them of shared values, and encourage optimism. Consider a quote, game or story. You might also use mindful silence to contemplate the themes to be addressed or mindful breathing to release tension. What will your opening be?

***Introduce Talking piece*** – Explain how the talking piece creates a space in which all participants can both speak and listen. The object used is typically something of meaning to the keeper or to the intent of the circle and can be passed easily from person to person.

- When you have the talking piece, you are invited to share. Otherwise, you’re invited to listen.
- Always an invitation to speak, so pass at any time.
- The keeper may speak without it to facilitate things but generally will not.

What talking piece will you use? How will you introduce its significance?

***Introduction/Check-in Round:*** Pose a question that invites participants get to know one another better, to build rapport and trust and to practice the use of the talking piece.

- What’s your name and a story about your name?
- What was the high or low point of your week?
- Share a story of a picture on your phone.

***Values/Guidelines*:** Participants in a circle play a major role in designing their own space by creating the values and guidelines for their discussion. The guidelines articulate the agreements among participants about how they will conduct themselves in the circle and cues behavior.

- Name one value that is important for you to be at your best in a group.
- If short on time, you may also introduce guidelines: respect the talking piece, honor confidentiality, speak/listen with openness and respect, stay in the circle.

What values question will you ask? Remember to mention any guidelines that don’t come up.

***Purpose*** + ***Storytelling Rounds:*** Remind participants of the purpose of the circle to prime them for the next rounds. Prompts encourage participants to share ***stories*** to increase understanding of one another. Give choices to allow for varying levels of vulnerability and progress increasingly deeper.

- What did you learn about conflict growing up?
- Share a story of someone who had an important influence on your life.
- Share a time when your values were affirmed or challenged at work.
- Share a story about the best team you’ve ever been a part of.

Craft 3-4 prompts that serve the purpose of your circle.

***Check-out Round:*** Pass the talking piece around and ask participants to share their thoughts about the circle.

- What are you taking from the circle today?
- What’s one word for how you feel leaving the circle today?

***Thanks & Closing:*** Thank everyone for coming and close the circle in a similar way to the opening: breathing, quote, reading, music, etc.
