# Supplementary material for: Restorative Community Building Practices: A Train-the-Trainer Workshop for Medical Students, Staff, and Faculty
Source: MedEdPORTAL. 2025 Sep 23;21:11547. doi: 10.15766/mep_2374-8265.11547 (PMC12454668; doi:10.15766/mep_2374-8265.11547)
Supplement: Supplementary file 1 — Training Schedule.docxRP Training Lecture 1.pptxRP Training Circle Scripts.docxRP in Academic Medicine.docxRP Training Lecture 2.pptxWorkshop Pre- and Postsurveys.docx3-Month Follow-Up Survey.docx [file mep_2374-8265.11547-s001.zip › F. Workshop Pre- and Postsurveys.docx]

**Appendix F: Pre- and Post- workshop surveys**

Purpose: This document is used to assess learner self-reported confidence in key skills the workshop aims to develop.

**Pre-workshop Survey**

Please indicate your level of agreement with the following statements. [ 1 = strongly disagree and 5 = strongly agree]

I can define restorative practices.

[Strongly disagree, disagree, neutral, agree, strongly agree]

I can describe general ways to apply restorative practices.

[Strongly disagree, disagree, neutral, agree, strongly agree]

I can envision applications of restorative practices within my practice/community.

[Strongly disagree, disagree, neutral, agree, strongly agree]

I feel confident enough to design a restorative practice activity/initiative within my practice/community.

[Strongly disagree, disagree, neutral, agree, strongly agree]

What are your expectations for the training today?

[Open Text]

**Post-workshop Survey**

Please indicate your level of agreement with the following statements.

I can define restorative practices.

[Strongly disagree, disagree, neutral, agree, strongly agree]

I can describe general ways to apply restorative practices.

[Strongly disagree, disagree, neutral, agree, strongly agree]

I can envision applications of restorative practices within my practice/community.

[Strongly disagree, disagree, neutral, agree, strongly agree]

I feel confident enough to design a restorative practice activity/initiative within my practice/community.

[Strongly disagree, disagree, neutral, agree, strongly agree]

How did the training compare to your expectations?

[Open Text]

What is the most important thing you learned today?

[Open Text]

What suggestions do you have for improvement?

[Open Text]
