# Supplementary material for: Restorative Community Building Practices: A Train-the-Trainer Workshop for Medical Students, Staff, and Faculty
Source: MedEdPORTAL. 2025 Sep 23;21:11547. doi: 10.15766/mep_2374-8265.11547 (PMC12454668; doi:10.15766/mep_2374-8265.11547)
Supplement: Supplementary file 1 — Training Schedule.docxRP Training Lecture 1.pptxRP Training Circle Scripts.docxRP in Academic Medicine.docxRP Training Lecture 2.pptxWorkshop Pre- and Postsurveys.docx3-Month Follow-Up Survey.docx [file mep_2374-8265.11547-s001.zip › G. 3-Month Follow-Up Survey.docx]

**Appendix G: 3-Month Follow-up Survey**

Purpose: This document is used to assess learner self-reported confidence with and perceptions of restorative practices 3 months after the workshop.

Please let us know if you agree or disagree with the following statements [1 = strongly disagree and 5 = strongly agree]

1. The training provided a solid foundation to apply restorative practices.
2. Circling promotes productive vulnerability.
3. Circling allows for meaningful perspective-sharing and story-telling.
4. Circling promotes a feeling of mutual support within the group.
5. Circling allows all members to participate equally.
6. Circling provides an opportunity to proactively address key problems or challenges my community is facing.
7. I would recommend learning restorative practices to a colleague.
8. I have been able to implement restorative practices.
9. Restorative practices have helped develop connections within my community.
10. Restorative practices have helped engage members of my community.
11. Restorative practices have improved my community’s ability to have meaningful conversations.
12. Restorative practices can minimize power dynamics.
13. Circling is an effective structure for discussions.
