# Supplementary material for: Restorative Community Building Practices: A Train-the-Trainer Workshop for Medical Students, Staff, and Faculty
Source: MedEdPORTAL. 2025 Sep 23;21:11547. doi: 10.15766/mep_2374-8265.11547 (PMC12454668; doi:10.15766/mep_2374-8265.11547)
Supplement: Supplementary file 1 — Training Schedule.docxRP Training Lecture 1.pptxRP Training Circle Scripts.docxRP in Academic Medicine.docxRP Training Lecture 2.pptxWorkshop Pre- and Postsurveys.docx3-Month Follow-Up Survey.docx [file mep_2374-8265.11547-s001.zip › _Educational Summary Report_11547.pdf]

MedEdPORTAL® The AAMC Journal of Teaching and Learning Resources

Original Publication Open Access

Restorative Community Building Practices: A Train-the-Trainer Workshop for Medical Students, Staff, and Faculty

Christopher L. Klasson*, Ada Gregory, MA, Ashrita Raghuram, Simran Sarin, Amal Shibli-Rahhal, MD, MS, MME

*Corresponding author: clklasson@uiowa.edu

Abstract

Introduction: : Restorative practices (RPs) are part of an emerging field that examines ways to build, strengthen, and manage relationships in organizations and communities, an approach that is gaining traction in the medical field. We describe the design and implementation of a day-long training for RPs focused on community-building at an academic medical institution that can be adapted at institutions interested in establishing a core of practitioners. **Methods:** We developed a day-long workshop to teach community-building practices to medical and physician assistant students, residents, staff, and faculty. The workshop included lectures, interactive activities, and group discussions. Participants completed pre- and postworkshop surveys and a 3-month follow-up survey to assess their understanding and application of RPs. Quantitative data were analyzed using a two-sample *t* test, and qualitative data were analyzed via content analysis. **Results:** The workshop had 25 attendees, with 92% and 88% response rates for pre- and postworkshop surveys, respectively. Participants reported significant improvements in their ability to define RPs, describe their applications, and design RP activities (all $p < .01$). Qualitative feedback highlighted benefits such as enhanced feelings of connectedness and trust. Three months later, feedback suggested that participants maintained positive perceptions of RPs and had increased confidence in applying them. **Discussion:** The workshop effectively developed RP capacity among participants, with positive indicators of sustained confidence in acquired skills. Limitations include the lack of longitudinal training for ongoing skill development and regular practice. Future work should explore longitudinal training models, advanced RP applications, and long-term follow-up to assess efficacy.

Keywords
Restorative Practices, Community Building, Community-Based Health Care

Educational Objectives

By the end of this activity, learners will be able to:

1. Define restorative practices.
2. List applications of restorative justice as a framework for building community.
3. Participate in hands-on restorative practice activities.
4. Develop a plan to apply lessons learned after the session.

Introduction

Restorative justice (RJ), an ethical approach emphasizing accountability and community connectedness, is evolving into a set of organizational processes called restorative practices (RPs) that are growing steadily in the medical field. These practices evolved from RJ efforts to reform the criminal justice system, using frameworks rooted in indigenous peacemaking processes intended to address wrongdoing and learn from past harms. A common RP centers on community-building to emphasize proactive connection and to prevent harm. According to Sawin and colleagues,[1] these practices can be applied to strengthen communities and establish and increase feelings of connection. RPs have shown promise in various education settings, including baccalaureate, undergraduate, medical, graduate training, and research environments.[2-5] Additionally, an academic medical institution has successfully used RPs as a foundation for conflict resolution within student affairs.[6]

One foundational RP is community-building circling (or circle[s]). Participants sit in a circle without any objects (i.e., tables) in the middle. A facilitator, known as the circle keeper, leads the group by asking scripted questions, referred to as prompts. A talking piece, which indicates the person allowed to speak, is passed around, giving each participant a turn to answer. Each prompt

Citation:
Klasson CL, Gregory A, Raghuram A, Sarin S, Shibli-Rahhal A. Restorative community building practices: a train-the-trainer workshop for medical students, staff, and faculty. *MedEdPORTAL*. 2025;21:11547. https://doi.org/10.15766/mep_2374-8265.11547

Copyright © 2025 Klasson et al. This is an open-access publication distributed under the terms of the Creative Commons Attribution license.

is answered by everyone in the circle, although individuals can choose not to respond, and this is called a round.[7] This structure promotes equitable conversations, allowing individuals to express their own needs and perspectives and to engage with the needs and perspectives of those around them. While these practices are based on foundational concepts, they are adaptable to the specific needs of the individuals and communities using them.

RP applications in academic medicine are being increasingly explored.[1] In this context, one of the main benefits of these practices is their consistent focus on creating equitable spaces and minimizing power dynamics, creating shared understandings to approach complicated issues, and establishing, maintaining/deepening, and repairing relationships. This makes these practices particularly useful tools to manage mistreatment and enhance the working and learning environment by promoting connection and civil discourse through storytelling. While different specific processes constitute RPs,[7,8] they all offer flexibility in application to meet individual community needs.

However, capacity building remains challenging for institutions looking to adopt RPs. National RP initiatives within academic medicine have been established in recent years, such as the AAMC facilitator training implemented since 2018,[9] and in-house courses offered at some institutions.[10]

At our institution, RPs were initially introduced by a trained student (Christopher L. Klasson), who collaborated with faculty and staff to implement community-building circling within various educational settings. Early projects included programming for undergraduate college students attending a summer enrichment program (the Summer Health Professions Education Program), a prematriculation program for first-year medical and physician assistant (PA) students, and a small-group activity for first-year medical and PA students.[11] Compared to previous iterations of the program, implementing RPs fostered earlier connection between students and encouraged meaningful self-reflection, which was valued by the students and program staff. These efforts increased interest in RPs among students, staff, and faculty, leading to the development of the RP training workshop described herein.

Methods

This training initiative consisted of a day-long workshop that included brief didactic components and focused on interactive engagement in RPs and RP activity design.

Facilitator Identification and Preparation

To implement the workshop, a sufficient number of trained facilitators was needed. We collaborated with an expert facilitator from another institution (Ada Gregory) to lead the training. This expert facilitator had experience in implementing RPs through prior ombudsperson work and had attended numerous trainings over a period of years. The session was cofacilitated by a medical student (Christopher L. Klasson), who had skills in RPs that were previously developed through training with Ada Gregory and refined through designing and implementing RPs at our institution. Facilitation assistance was provided by a second medical student (Ashrita Raghuram), who was previously trained in community-building practices through a prior RP project at our institution.[11] Her training involved participating in a community-building circle, followed by receiving instruction on how to facilitate a circle and then facilitating introductory circles under supervision. This specific training was focused on facilitation techniques and did not include activity design, and therefore Ashrita Raghuram's role in this workshop was to facilitate a predesigned circle.

Participants

The workshop was offered to medical and PA students, staff, and faculty at our institution. Enrollment was achieved via faculty and student email lists as well as word-of-mouth. Participation was voluntary. The University of Iowa Institutional Review Board (No. 202403636) deemed this project exempt on April 7, 2024.

Learning Environment

The workshop was delivered in-person over 9 hours on a single day, including a 30-minute breakfast and a 1 hour-long lunch with food and drink provided. No preliminary work was required. The desired ratio of facilitators to attendees was set at 1:10 to promote familiarity among participants and keep group sizes manageable for circling activities (see Appendix A for the workshop schedule).

Educational Content

The workshop's content was developed by the two primary facilitators (Ada Gregory and Christopher L. Klasson), except for the affective statements as well as the cited images.[12,13] The focus was on experiencing, planning, and executing community-building circles, with additional time for group discussion and debriefing. Lecture time was limited to key information; the didactic component discussed core RJ principles, and RP applications within medicine.

The workshop started with an introductory lecture to RJ theory and core concepts in RPs (Appendix B). After the lecture,

Copyright © 2025 Klasson et al. This is an open-access publication distributed under the terms of the Creative Commons Attribution license.

attendees participated in a circle where circling rules were explained. The initial portion of the circle was completed by all attendees as one large group (Appendix C, pages 1-2). Each participant shared their name and key values with the whole group. Participants were then randomly assigned into three smaller groups, each facilitated by a trained facilitator who led a full community-building circle (Appendix C, page 3). This was followed by a debriefing session to explore participants' reactions to the circling process and discuss the benefits of establishing a restorative culture in the learning environment and workplace. This discussion was facilitated by the lead workshop facilitators. After this debrief, trainers led participants through a discussion of restorative approaches and language centered around affective statements and questions, exploring different ways these practices can be implemented into working and learning cultures.

Participants were then divided into groups of three individuals, and each group was asked to design a circle script using a step-by-step instruction worksheet (Appendix D), with assistance from the lead facilitators (Ada Gregory and Christopher L. Klasson) as needed. Groups of three individuals were then combined into a larger group of six individuals. The larger groups conducted two circles, using the scripts developed by the two smaller groups. The two circles were led by the group members themselves (a circle keeper was chosen from each smaller group) and were separated by a short debrief. The workshop facilitators were available for general guidance.

A second brief lecture discussed the benefits and reasons for implementing RPs within medicine (Appendix E). To conclude the day, attendees drafted their own RP interventions using the provided worksheet with templated guidance and facilitators available for assistance.

Assessment

Prior to the workshop, demographic information such as participant role within the organization (student, staff, resident, faculty) was collected in the workshop sign-up form.

Participants were invited to complete voluntary surveys before and after the workshop. Surveys were developed by the study team without specifically referring to any source, due to lack of existing literature describing similar workshops in academic medicine. There was no pilot testing or validity evidence of these surveys prior to use for this workshop. The preworkshop survey consisted of four questions, with participant responses rated on a 5-point Likert scale (1 = *strongly disagree*, 5 = *strongly agree*), assessing preexisting knowledge of RPs and the ability to envision RP applications. The survey included one open-ended question about expectations for the workshop (Appendix F, page 1). The postworkshop survey consisted of the same closed-ended questions and three open-ended questions addressing how the training met expectations, the most important concept learned, and suggestions for improvement (Appendix F, page 2).

Participants were invited to enroll in a follow-up study that included a survey administered 3 months after the training date to assess changes in perceptions and attitudes toward RPs over time. The follow-up survey consisted of 13 questions regarding preparedness to implement RPs, perceptions of RPs, and ability to enact RPs in their communities (Appendix G). These questions were answered using a 5-point Likert scale (1 = *strongly disagree*, 5 = *strongly agree*). A response of not applicable (N/A) was an option if participants felt they did not have adequate experience to answer a particular question. All surveys were administered through Qualtrics.

Data Analysis

Because of the small number of participants and the data distribution, quantitative data were analyzed in R studio using a nonparametric Wilcoxon rank-sum test. Responses to the preworkshop, postworkshop, and 3-month follow-up surveys are expressed as mean ± standard deviation ratings, and as the percentage of respondents who chose *agree* or *strongly agree* in response to survey questions. In the 3-month follow-up survey analysis, N/A responses were subtracted from the denominator when calculating the percentage of *agree*/*strongly agree* responses, and therefore these data were not counted in the mean score calculations. Statistical significance was defined as a p value of less than .05.

Qualitative data were analyzed via content analysis with consensus coding. Two researchers (Christopher L. Klasson and Simran Sarin) independently coded each response with relevant themes. Multiple codes could be applied to an individual response. Themes were summarized in a codebook with similar thematic categories consolidated into a common theme. The two sets of coded responses were compared, and disagreements were resolved to obtain a final consensus coding. Themes were included if they were present in more than 10% of responses for an individual question.

Results

Learner Characteristics and Survey Response Rates

This workshop was delivered in May 2024, with 25 attendees: nine medical students, one PA student, nine faculty members involved in medical education and the College of Public Health,

Copyright © 2025 Klasson et al. This is an open-access publication distributed under the terms of the Creative Commons Attribution license.

five staff members across three separate departments, and one resident.

Completion of the pre- and postworkshop surveys was optional, and each survey was administered separately. There were 23 preworkshop survey responses (92%) and 22 postworkshop responses (88%). Self-identified personal characteristics of the respondents were not collected, to protect anonymity.

Program Evaluation: Immediate Pre- and Post Workshop Surveys
Quantitative responses: Pre- and postworkshop responses to the four closed-ended questions are shown in Table 1. The preworkshop responses indicated limited knowledge and confidence regarding RPs. On the postworkshop survey, the majority of respondents indicated that they understood RPs and their applications, and felt confident in designing an RP activity. The difference between pre- and postworkshop mean ratings was significant across every survey question.

Qualitative responses: On the preworkshop survey, most respondents indicated an interest in learning about RPs and applying them. On the postworkshop survey, participants indicated that the workshop met or exceeded their expectations. Feelings of connectedness were consistently noted by participants. Suggestions for improvement primarily focused on structural adjustments to the workshop, while 67% of responses noted that no changes were necessary (Table 2).

Program Evaluation: 3-Month Follow-Up Survey
Eleven participants agreed to participate in the follow-up study and completed the 3-month survey (100% response rate). These 11 participants responded to all items on the follow-up survey. Participants included six students, two faculty members, and three staff. Overall, respondents indicated continued positive perceptions of RPs and confidence in applying them, as well as the belief that RPs can prove beneficial for communities (Table 3).

Discussion

We developed a day-long workshop to teach RPs for community-building to medical and PA students, residents, staff, and faculty. Postworkshop, participants showed high confidence in their understanding of RJ and its applications, feeling equipped to design their own RP interventions.

Participants identified specific benefits from RPs, notably feelings of connection and trust. They also provided useful insights for improving future workshops, such as adjusting the way groups are arranged, primarily making them smaller and rotating participants throughout the day to promote exposure to different attendees. We adopted this recommendation for later iterations and suggest that it be implemented in similar trainings.

Trainers also received anecdotal comments from several attendees expressing a desire for inclusion of material targeting conflict resolution. While conflict resolution presents an important application of RPs, training on conflict resolution is a more advanced topic that is beyond the scope of this time-limited, introductory-level workshop. However, one can envision a series of future workshops that span several more advanced RP applications, including conflict resolution.

Responses to the 3-month follow-up survey indicated that participants continued to feel prepared to apply RPs, thereby supporting the workshop's ability to develop RP capacity. In particular, we believe that the participants' ongoing belief that RPs can promote connectedness is particularly meaningful, as a lack of feelings of belonging is known to be associated with poor organization retention and increased risk for burnout among students.[14,15] However, fewer than 50% of the original

Table 1. Participant Responses on Pre- and Postworkshop Surveys Regarding Understanding of and Confidence in Applying Restorative Practices

Survey Questions	Preworkshop Survey (N = 23)		Postworkshop Survey (N = 22)	
	M (SD)[a]	Agree/Strongly Agree %	M (SD)[a]	Agree/Strongly Agree %
I can define restorative practices.	2.95 (1.06)	43	4.40[b] (0.50)	100
I can list general ways to apply restorative practices.	2.82 (1.15)	35	4.54[b] (0.91)	95
I can envision applications of restorative practices within my practice/community.	3.30 (1.25)	43	4.72[b] (0.63)	91
I feel confident enough to design a restorative practice activity/initiative within my practice/community.	2.00 (1.34)	17	4.54[b] (0.59)	95

[a]Scored on a 5-point Likert scale (1 = *strongly disagree*, 5 = *strongly agree*).
[b]$p < .01$ between pre- and postworkshop survey mean ratings.

Copyright © 2025 Klasson et al. This is an open-access publication distributed under the terms of the Creative Commons Attribution license.

Table 2. Participant Responses to Pre- and Postworkshop Qualitative Survey Questions and Thematic Analysis

Questions	No. of Responses	Themes (% of Coded Responses)	Representative Quotes
Preworkshop Question			
"What are your expectations for today's training?"	23	*Learning about RP (65%):* Desire to attend the training to either establish or advance understanding of RPs.	"Better understanding of principles and application of restorative justice."
		Application of RP (43%): Attending the training with the specific aim of equipping oneself to apply RPs.	"Hoping to come away with tangible tools to embody and spread restorative justice principles"
Postworkshop Questions			
"How did the training compare to your expectations?"	22	*Met expectations (95%):* Indicated that the training met or exceeded their expectations.	"Better than I had expected. Gave me concrete skills to attempt incorporating principles of restorative justice in my daily interactions and in my teaching and research activities."
		Fostered connection (22%): Responses indicating that participants felt connected to other attendees.	"I got to know people in my community that I would have never gotten to know otherwise."
		Effective structure (14%): Responses addressing the positive impact of the training design on learning.	"I am so glad there were so many opportunities to apply the RJ practices and circle development."
"What is the most important thing you learned today?"	22	*Skill Acquisition (50%):* Responses indicating feeling of preparedness to apply RPs with skills learned at the workshop, which includes envisioning potential applications and technical skills.	"How to create space to understand different perspectives and subsequently develop the capacity to empathize."
		Social connection (41%): Responses remarking upon learning the degree to which RPs can promote feelings of connection.	"I am taking away a renewed understanding of the critical importance of trust and relationship for healing, ability to work together, innovation."
"What suggestions do you have for improvement?"	22	*Changes to format (27%):* Responses provided suggestions regarding the format of the workshops or some of its components.	"Mix up the groups more—instead of a 6-person group with 2 subgroups that reconvene, have the 3-person groups stay together but then mix up them up after the first reflection"

Abbreviations: RPs, restorative practices; RJ, restorative justice.

participants participated in the follow-up survey, and therefore these results may not be reflective of the entire group. Findings from this follow-up survey should be investigated in a larger and longer study.

This workshop has several limitations. The lack of longitudinal training precludes opportunities for repeated practice and feedback, and the introduction of more advanced RP applications and the small number of participants in the 3-month follow-up survey reduces the ability to assess the long-term impact of the training. The lack of pilot or validity testing for the survey instruments used may limit the accuracy of measurements used to assess the efficacy of this workshop.

An additional potential limitation is the inclusion of students, staff, and faculty in the same workshop, as the different groups likely have different needs in relation to their application of RPs. However, mixing the groups did come with the practical benefit

Table 3. Participant Responses to 3-Month Follow-Up Survey Questions ($N = 11$)

Questions	*M (SD)*[a]	Agree/Strongly Agree %	No. of N/A Responses
The training provided a solid foundation to apply restorative practices.	4.64 (0.50)	100	0
Circling promotes productive vulnerability.	4.73 (0.46)	100	0
Circling allows for meaningful perspective-sharing and storytelling.	4.73 (0.46)	100	0
Circling promotes a feeling of mutual support within the group.	4.82 (0.40)	100	0
Circling allows all members to participate equally.	4.64 (0.40)	91	0
Circling provides an opportunity to proactively address key problems or challenges my community is facing.	4.55 (0.84)	73	1
I would recommend learning restorative practices to a colleague.	4.82 (0.40)	100	0
I have been able to implement restorative practices.	4.18 (0.94)	73	1
Restorative practices have helped develop connections within my community.	3.91 (0.83)	82	0
Restorative practices have helped engage members of my community.	4.27 (0.78)	73	2
Restorative practices have improved my community's ability to have meaningful conversations.	4.27 (1.16)	45	2
Restorative practices can minimize power dynamics.	4.55 (0.68)	91	0
Circling is an effective structure for discussions.	4.73 (0.46)	100	0

[a]Scored on a 5-point Likert scale (1 = *strongly disagree*, 5 = *strongly agree*).

Copyright © 2025 Klasson et al. This is an open-access publication distributed under the terms of the Creative Commons Attribution license.

of exhibiting how RPs can be used to mitigate power dynamics as students worked alongside and connected with faculty. Similarly, only one resident participated, which restricts insight into how RPs are perceived by graduate medical learners.

Due to the voluntary nature of the training, selection bias may be present, since it included participants inclined toward or with previous exposure to RPs. This in turn may have increased the likelihood that participants would reflect positively on the experience. Likewise, the academic environment where this workshop was delivered may limit generalizability to other medical settings such as community or federal institutions.

Finally, this training necessitates the development of an initial cadre of trainers, likely through commercially available programs, representing a substantive financial and time commitment. While there is no universal training standards established within academic medicine for independently implementing RPs, this workshop, as well as the several days–long AAMC training program, may provide a starting point.[9] Future work should include creating guidance for institutions regarding the level of training needed for various levels of RPs. Subsequent scaling of local training may need to be gradual as new facilitators gain confidence and experience. Finally, the day-long format also requires a significant time and resource commitment from participants and their departments/units. An alternative format could include longitudinal courses with participants meeting at multiple times in shorter class sessions to cover the same material.

Medical education and practice environments need tools to bolster a culture that supports connectedness and dialogue. RPs offer a promising tool for those needs. This workshop provides a format for local training of practitioners in RPs for community-building. Future work should explore approaches that would maintain a cycle of training, introduce more advanced applications, and conduct long-term follow-up to better assess training efficacy and overall benefits to the individuals and institutions.

Appendices

A. Training Schedule.docx
B. RP Training Lecture 1.pptx
C. RP Training Circle Scripts.docx
D. RP in Academic Medicine.docx
E. RP Training Lecture 2.pptx
F. Workshop Pre- and Postsurveys.docx
G. 3-Month Follow-Up Survey.docx

All appendices are peer reviewed as integral parts of the Original Publication.

Christopher L. Klasson: Medical Student, University of Iowa Roy J. and Lucille A. Carver College of Medicine; ORCID: https://orcid.org/0000-0003-1250-150X

Ada Gregory, MA: Associate Director, Kenan Institute of Ethics, Duke University

Ashrita Raghuram: Medical Student, University of Iowa Roy J. and Lucille A. Carver College of Medicine

Simran Sarin: Medical Student, University of Iowa Roy J. and Lucille A. Carver College of Medicine

Amal Shibli-Rahhal, MD, MS, MME: Associate Dean for Student Affairs and Curriculum, University of Iowa Roy J. and Lucille A. Carver College of Medicine; Clinical Professor of Endocrinology and Metabolism, Department of Internal Medicine, University of Iowa Roy J. and Lucille A. Carver College of Medicine

Disclosures
None to report.

Funding/Support
None to report.

Ethical Approval
The University of Iowa Institutional Review Board reviewed this project.

References

1. Sawin G, Klasson CL, Kaplan S, et al. Scoping review of restorative justice in academics and medicine: a powerful tool for justice equity diversity and inclusion. *Health Equity*. 2023;7(1):663-675. https://doi.org/10.1089/heq.2023.0071
2. Shetty N, Pierson E, Manders KB, Sun Y, Masciantonio E, Sinko L. Building a trauma-informed community: applying community building circles to an academic health research setting. *Acad Med*. 2024;99(2):164-168. https://doi.org/10.1097/ACM.0000000000005491
3. Jaochico MS. *A Phenomenological Study of the Circle Experience: Restorative Justice in a Graduate Academic Environment*. University of West Georgia; 2023. Accessed August 15, 2025. https://www.proquest.com/docview/2845356107/abstract/8C4E989BC9884BA6PQ/1
4. Acosta D, Karp DR. Restorative justice as the Rx for mistreatment in academic medicine: applications to consider for learners,

Copyright © 2025 Klasson et al. This is an open-access publication distributed under the terms of the Creative Commons Attribution license.

faculty, and staff. *Acad Med*. 2018;93(3):354-356. https://doi.org/10.1097/ACM.0000000000002037

5. Mohama A, Shih S, Behel J. From compassion fatigue to connection: the role of story and art in healthcare. *J Gen Intern Med*. Published online May 12, 2025. https://doi.org/10.1007/s11606-025-09587-3
6. Behel JM, Israni ST. Restorative practice in healthcare: case studies from academic medicine. In: Roth KR, Kumah-Abiwu F, Ritter ZS, eds. *Restorative Justice and Practice in US Education*. Palgrave MacMillan; 2024:201-215.
7. Pranis K. *The Little Book of Circle Processes: A New/Old Approach to Peacemaking*. Simon and Schuster; 2015.
8. Moore DB, Vernon A. *Setting Relations Right in Restorative Practice: Broadening Mindsets and Skill Sets*. Routledge; 2023.
9. Restorative justice in academic medicine facilitator training. Accessed August 15, 2025. https://cloud.email.aamc.org/RPAM
10. Gallagher S. Using restorative justice to build a culture of belonging & continuous growth. Duke University School of Medicine. December 9, 2024. Accessed August 15, 2025. https://psychiatry.duke.edu/news/using-restorative-justice-build-culture-belonging-continuous-growth
11. Klasson CL, Shibli-Rahhal A, Ortiz E, et al. Restorative justice practices as a foundation for medical education innovation. Clin Teach. 2025;22(1):e13852.
12. Schein EH, Schein PA. *Humble Inquiry: The Gentle Art of Asking Instead of Telling*. 2nd ed. Berrett-Koehler Publishers; 2021.
13. Rosenberg MB. *Nonviolent Communication: A Language of Life: Life-Changing Tools for Healthy Relationships*. 3rd ed. PuddleDancer Press; 2015.
14. Leep Hunderfund AN, Saberzadeh Ardestani B, Laughlin-Tommaso SK, et al. Sense of belonging among medical students, residents, and fellows: associations with burnout, recruitment retention, and learning environment. *Acad Med*. 2025;100(2):191-202. https://doi.org/10.1097/ACM.0000000000005892
15. Silver EM, Balas K, Ellinas EH, et al. Sense of belonging and intent to leave among medical school faculty. *JAMA Netw Open*. 2025;8(4):e257728. https://doi.org/10.1001/jamanetworkopen.2025.7728

Received: April 6, 2025
Accepted: August 11, 2025
Published: September 23, 2025

Copyright © 2025 Klasson et al. This is an open-access publication distributed under the terms of the Creative Commons Attribution license.
